# Pivoting of microtubules driven by minus-end-directed motors leads to spindle assembly

**DOI:** 10.1186/s12915-019-0656-2

**Published:** 2019-05-23

**Authors:** Lora Winters, Ivana Ban, Marcel Prelogović, Iana Kalinina, Nenad Pavin, Iva M. Tolić

**Affiliations:** 1 Max Planck Institute of Molecular Cell Biology and Genetics, Pfotenhauerstr. 108, 01307 Dresden, Germany; 2 Department of Physics, Faculty of Science, University of Zagreb, Bijenička cesta 32, 10000 Zagreb, Croatia; 3 Division of Molecular Biology, Ruđer Bošković Institute, Bijenička cesta 54, 10000 Zagreb, Croatia

**Keywords:** Mitotic spindle, Mitosis, Spindle assembly, Antiparallel microtubule bundles, Overlap bundles, Microtubule rotation, Motor proteins, Kinesin-5, Forces, Fission yeast

## Abstract

**Background:**

At the beginning of mitosis, the cell forms a spindle made of microtubules and associated proteins to segregate chromosomes. An important part of spindle architecture is a set of antiparallel microtubule bundles connecting the spindle poles. A key question is how microtubules extending at arbitrary angles form an antiparallel interpolar bundle.

**Results:**

Here, we show in fission yeast that microtubules meet at an oblique angle and subsequently rotate into antiparallel alignment. Our live-cell imaging approach provides a direct observation of interpolar bundle formation. By combining experiments with theory, we show that microtubules from each pole search for those from the opposite pole by performing random angular movement. Upon contact, two microtubules slide sideways along each other in a directed manner towards the antiparallel configuration. We introduce the contour length of microtubules as a measure of activity of motors that drive microtubule sliding, which we used together with observation of Cut7/kinesin-5 motors and our theory to reveal the minus-end-directed motility of this motor in vivo.

**Conclusion:**

Random rotational motion helps microtubules from the opposite poles to find each other and subsequent accumulation of motors allows them to generate forces that drive interpolar bundle formation.

**Electronic supplementary material:**

The online version of this article (10.1186/s12915-019-0656-2) contains supplementary material, which is available to authorized users.

## Background

During cell division, the genetic material is divided into two equal parts by the mitotic spindle. This complex dynamic micro-machine is made of microtubules (MTs) emanating from the spindle poles, chromosomes, and a variety of accessory proteins [1, 2]. Some MTs extending from the spindle pole are bound to kinetochores on the chromosome, whereas others are bound to MTs extending from the opposite pole, in an antiparallel configuration known as interpolar or overlap bundles [3–6]. These bundles interact laterally with kinetochore MTs and regulate the forces acting on chromosomes and spindle poles [7–12].

MTs within interpolar bundles are crosslinked by specific proteins, which can be divided into three classes: (i) motors that slide the MTs and thus the spindle poles apart by walking along the MTs away from the pole, i.e., towards the plus end of the MT, such as kinesin-5 motors Cut7/Cin8/Eg5/KIF11 [13–15]; (ii) motors that pull the poles together by walking along the MTs towards the pole, i.e., towards the minus end of the MT, such as kinesin-14 motors Ncd/HSET/KifC1 [16, 17]; and (iii) proteins that crosslink MTs without walking along the MTs, such as Ase1/PRC1 [18]. Remarkably, in vitro studies have shown that kinesin-5 motors can also move towards the minus end of the MTs, when walking on a single MT or in a non-crowded environment on antiparallel MTs [19–23]. Likewise, kinesin-14 motors can reverse the direction of movement under a low external force [24]. Stability of antiparallel bundles for combinations of motors and crosslinkers has been explored theoretically [25, 26].

While the already formed antiparallel bundles in metaphase have been to a large extent described, little is known about how these highly organized structures are formed during prometaphase. The reason is that this dynamic process is not accessible by current experimental techniques due to a high number of MTs extending from the spindle poles in prometaphase in higher eukaryotic cells [27], which may form antiparallel bundles. In yeast cells, which have a small number of MTs and a rod-shaped spindle [4, 6], the study of the antiparallel bundle formation in living cells is challenging because the spindle poles are next to each other at the onset of prometaphase. Yet, the advantage of yeasts as experimental systems is that their spindles consist of only one antiparallel bundle. Electron tomography on early spindles in yeast showed MTs interacting at oblique angles [28], suggesting that such interactions may be an intermediate step during the formation of antiparallel bundles.

Alignment of MTs into an antiparallel configuration may be achieved by rotation of MTs that initially extend at an oblique angle. Indeed, live-cell imaging in fission yeast showed that MTs change their angle as they rotate (i.e., pivot) around the spindle pole [29]. Eventually, these MTs join the spindle, with help from Ase1 crosslinkers [30]. Experiments on budding yeast showed that cells lacking kinesin-14 motors have more MTs extending at an oblique angle with respect to the spindle and fewer antiparallel MTs than wild-type cells do [31]. Based on this finding, the authors hypothesized that minus-end-directed motors align the MTs, which was verified by computer simulations [31]. Spindle assembly starting from a monopole has been explored by extensive computer simulations including motors of different directionalities and passive crosslinkers [32]. Experiments together with simulations showed that spindles can form even in the absence of motors, in which case MT growth and Ase1 crosslinkers play an important role [33–35]. Even though the formation of antiparallel bundles has been explored theoretically, a direct observation of interpolar bundle formation in vivo is missing.

## Results

### Assay for spindle reassembly in fission yeast

**Fig. 1:**
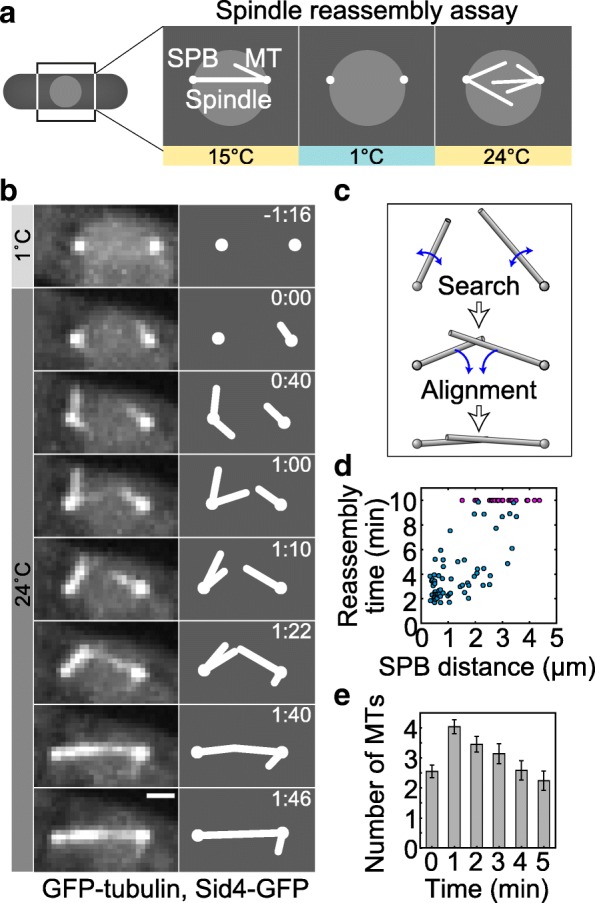
Spindles reassemble by rotational movements of MTs. **a** Spindle reassembly assay. Mitotic cells were cooled to 1 °C to depolymerize MTs (Methods). Once the temperature was increased to 24 °C, MTs grew from the SPBs and reassembled the spindle. **b** Time-lapse images of spindle reassembly in a wild-type cell expressing GFP-tubulin and Sid4-GFP (strain KI061). Images are maximum-intensity projections, time is given in min:s, scale bar, 1 μm. Corresponding schemes are shown to the right. **c** Scheme showing how the formation of an antiparallel MT bundle occurs in two steps. SPBs are represented as spheres and MTs as rods. **d** Reassembly time, defined as the time from the onset of MT growth until the formation of the antiparallel MT bundle (spindle) between the SPBs, as a function of the distance between the SPBs at the onset of MT growth. *n* = 87 cells; pink data points denote cells in which the spindle was not reassembled within 10 min. **e** Number of polar MTs per cell during the first 5 min following the onset of MT growth, *n* = 28 cells, error bars, s.e.m.

At the onset of mitosis in the fission yeast *Schizosaccharomyces pombe*, the two spindle pole bodies (SPBs) are embedded in the nuclear envelope, which remains intact during mitosis [36]. The SPBs nucleate polar MTs with minus ends at the SPBs and the plus ends in the nucleoplasm [4, 37]. MTs extending from the opposite SPBs interact and form an antiparallel interpolar bundle and together with MTs that bind to kinetochores assemble the spindle. The interactions between antiparallel MTs occur when the SPBs are next to each other [38], making it difficult to study the dynamics of this process. To increase the distance between the SPBs, we used a spindle reassembly assay, in which we disassembled the spindle by exposing the cells in metaphase to cold temperature (1 **°**C, Fig. 1a; Additional file 1: Figure S1a), adapting the approach that was previously used to study kinetochore capture [29, 39]. SPBs were visualized by Sid4-GFP and MTs by GFP-tubulin. When the temperature was increased to permissive temperature (24 °C), the SPBs were more than 1 μm apart in 61 ± 5% of cells (*n* = 84; results are mean ± s.e.m. unless otherwise stated) (Additional file 1: Figure S1b). Thus, this assay allowed us to investigate the process of antiparallel bundle formation, i.e., spindle reassembly.

### Antiparallel microtubule bundles are formed in two steps

After the cold treatment was ended and the cells returned to permissive temperature, 75 ± 5% (65 out of 87) spindles reassembled within 10 min, which is a typical duration of prophase and metaphase in unperturbed mitosis [40]. Shortly after the return to permissive temperature, MTs started growing from each of the two SPBs (defined as time 0, Fig. 1b). The structure that appears as a MT is likely a bundle of a few MTs, but because they move as a single object, we will refer to them simply as a MT. MTs did not extend in a defined direction, but instead pivoted around the SPB (Fig. 1b, 0:00–1:22), in agreement with our previous observations [29]. Eventually, a MT extending from one SPB came into contact with a MT from the other SPB (Fig. 1b, 1:22). At the time of initial contact, MTs were typically not aligned in an antiparallel manner, but interacted at an oblique angle (Fig. 1b, 1:22). In 21 out of 31 cells that reassembled their spindles and had the SPBs separated by more than 1 μm, MTs interacted at an oblique angle, in 6 they met at the pole-pole axis and in 4 one MT grew directly to the opposite pole. Following the initial contact, MTs rotated into antiparallel alignment (Fig. 1b, 1:22–1:46, Additional file 2: Movie S1; note that not every apparent contact leads to alignment). Thus, formation of an antiparallel bundle occurs in two steps: (i) MT growth and random rotation before their contact, which we refer to as search, and (ii) directed rotation of MTs towards an antiparallel configuration, which we term aligning (Fig. 1c).


**Additional file 2: Movie S1.** Spindle reassembly in an *S. pombe* cell expressing GFP-tubulin and Sid4-GFP (strain KI061). Images are maximum-intensity projections; time is given in min:s starting from time 0 when microtubules start to grow, scale bar, 1 μm. Time 0 is the time when the cold treatment ended and the temperature was raised to 24 °C. The movie corresponds to Fig. 1b. (MP4 1350 kb)


### Quantification of spindle reassembly

To quantify the kinetics of spindle reassembly, we measured the spindle reassembly time, defined as the time needed for the formation of an antiparallel MT bundle between the SPBs, which includes both steps of this process. The average reassembly time was 7.3 ± 0.9 min (*n* = 87 cells). The cells with a larger initial distance between the SPBs took a longer time to reassemble the spindle or did not reassemble the spindle within 10 min (Fig. 1d).

To describe the first step of bundle formation, in which MT contact is established, we quantify polar MTs and their movement. During the process of spindle reassembly, the average number of polar MTs per cell increased from 0 to 2.5 during the first minute and to 4 in the second minute (*n* = 28 cells, Fig. 1e). Afterwards, the number of MTs decreased. MTs typically reached a length of 1.5 μm and spent most of their lifetime at a rather constant length (Additional file 1: Figure S1c), whereas their angular diffusion coefficient was 4.5°^2^/s (Additional file 1: Figure S1d), in agreement with our previous measurements [29]. Note that, because the structure that appears as a polar MT is likely a bundle of a few MTs, our measurements correspond to the bundle.

### Microtubules slide sideways towards each other’s minus end during the process of alignment

**Fig. 2:**
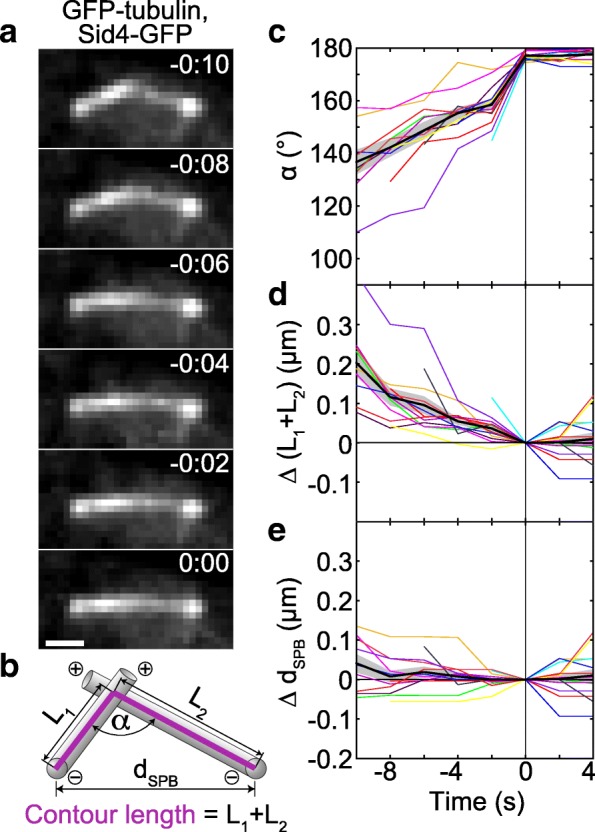
MTs slide sideways towards each other’s minus end during alignment. **a** Time-lapse images of a wild-type cell expressing GFP-tubulin and Sid4-GFP (strain KI061) during the formation of an antiparallel bundle. Images are maximum-intensity projections, time is given in min:s, scale bar, 1 μm. **b** Measurement of the angle between the MTs, *α*, the contour length of MTs, *L*_1_ + *L*_2_, and the distance between the SPBs, *d*_SPB_. The contour length of the MTs is defined as a segmented line (magenta) starting at one SPB, passing through the contact point between the MTs, and ending at the other SPB. SPBs are represented as spheres and MTs as rods; plus and minus signs designate the respective ends of MTs. **c** Angle between MTs, *α*, as a function of time. **d** Contour length difference as a function of time. The contour length difference is defined as the difference between the contour length, *L*_1_ + *L*_2_, at a given time and the contour length at *t* = 0, Δ(*L*_1_ + *L*_2_) = *L*_1_ + *L*_2_ − (*L*_1_ + *L*_2_)|_*t* = 0_. **e** The difference of distances between the SPBs as a function of time, defined as the difference between the SPB distance, *d*_SPB_, at a given time and the distance at *t* = 0, Δ*d*_SPB_ = *d*_SPB_ − *d*_SPB_|_*t* = 0_. In panels **c**–**e**, the same strain as in **a** was used; time 0 is the time when the antiparallel bundle was formed; *n* = 14 cells; individual cells (colored lines), mean value (black line), and s.e.m. (shaded area) are shown

In the second step of bundle formation, MTs rotate into antiparallel alignment by sliding sideways along each other towards the SPB (Fig. 2a). To quantify the geometry of this system over time, we first measured the angle between the MTs, *α* (Fig. 2b), starting 10 s before the antiparallel bundle was formed. We found that the angle increased towards 180°, which represents the bundled configuration (Fig. 2c). During the subsequent 4 s, the angle remained constant (Fig. 2c).

Sideways sliding of MTs towards antiparallel alignment may be driven by motors at the MT contact point. To assess the direction and velocity of the movement of these motors, we introduce a measure termed the contour length of the MTs. We define the contour length of the MTs as a segmented line starting at one SPB, passing through the contact point between the MTs, and ending at the other SPB (Fig. 2b; Additional file 1: Figure S1e; Methods). Note that, due to limitations in spatial resolution, we cannot determine whether this contact point is at the MT end or at the side of the MT. Thus, we use sideways sliding as a more general interpretation of the observed movement. We found that the contour length of the MTs decreased during the rotation of the MTs into antiparallel alignment and remained constant afterwards (Fig. 2d). The contour length decreased in a roughly linear manner, which allowed us to introduce the measure termed contour velocity, defined as the velocity at which the contour length changes. We measured a contour velocity of − 18 ± 2 nm/s (*n* = 14 cells). Contrary to the contour length, the distance between the SPBs was constant both during the alignment and afterwards (Fig. 2e). Given that MTs do not undergo poleward flux in fission yeast [41], our results reveal that during the alignment the contact point between the MTs moves in a directed manner towards the minus end of each MT, which is at the SPB. Thus, the alignment may be driven by minus-end-directed motors. The velocity at which the motors slide the MTs with respect to each other equals the contour velocity. If these motors are dimeric and walk along one MT, while their tail is attached to the other MT, their velocity is roughly 20 nm/s. On the other hand, if the motors are tetrameric and walk along both MTs simultaneously, their velocity along each MT is roughly 10 nm/s.

### Cut 7 (kinesin-5) is important for spindle reassembly

We explored the role of candidate motor proteins and a non-motor MT crosslinker in spindle reassembly. First, we focused on Cut7, a kinesin-5 family member, because it is essential for spindle formation and localizes to the spindle midzone and poles [13, 42]. Moreover, Cut7 can move towards the minus end of the microtubule in vitro [20], suggesting that it can drive MT alignment. On the other hand, spindles are able to assemble without any of the other eight kinesins in *S. pombe* [43–48], dynein [49], and the non-motor crosslinker Ase1/PRC1 [50, 51].

**Fig. 3:**
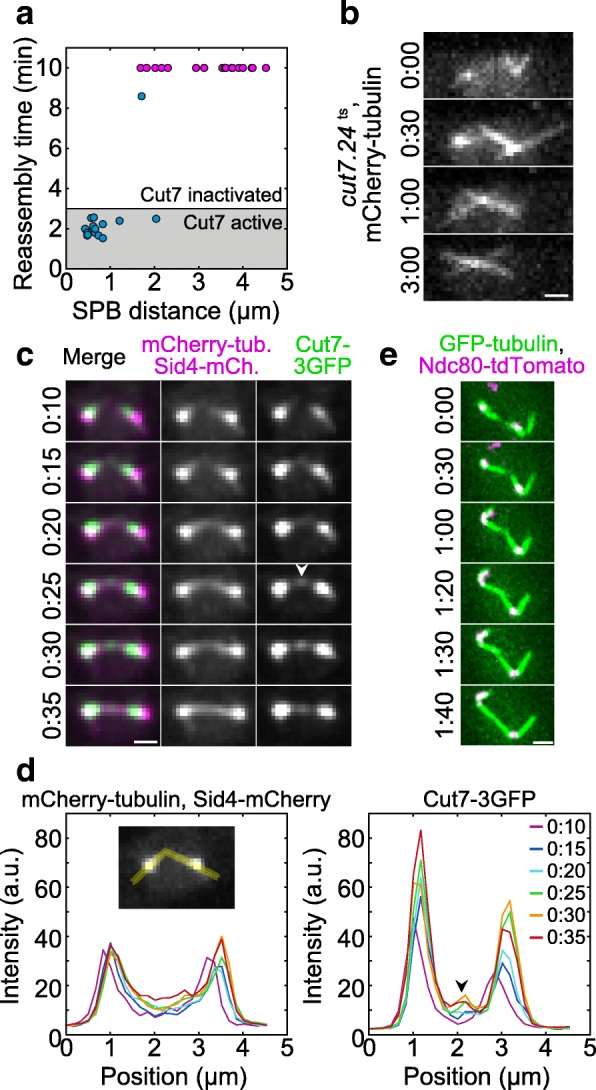
Cut7-GFP is found at the initial contact points of MTs. **a** Reassembly time as a function of the distance between the SPBs for cut7.24^ts^ cells (strain CF.391) at non-permissive temperature (37 °C), *n* = 34 cells. Pink data points denote cells in which the spindle was not reassembled within 10 min. **b** Time-lapse images of a cut7.24^ts^ cell expressing mCherry-tubulin (strain CF.391) at non-permissive temperature (37 °C), in which the spindle did not reassemble. **c** Spindle reassembly in a cell expressing Cut7-3GFP (green), mCherry-tubulin (magenta), and Sid4-mCherry (magenta; strain LW042). Merged time-lapse images (left column) and separate channels (central and right column, both in gray scale) are shown. Note the accumulation of Cut7 at the site of MT contact (arrowhead). **d** Signal intensity profiles of mCherry-tubulin (left) and Cut7-3GFP (right) measured along the MT contour extended into the cytoplasm (see example in the inset on the left), at times in min:s noted in the legend on the right (strain LW042). The arrowhead marks the peak of Cut7-3GFP signal in the MT contact region. The intensity profiles were measured on the images shown in panel **c**. **e** Spindle reassembly in a cell expressing GFP-tubulin (green) and Ndc80-tdTomato (a kinetochore marker, magenta; strain AH01). Note that spindle reassembly including MT alignment occurs without kinetochores being present close to the MT contact point. In these experiments, cold treatment was performed as described in [29], and images were acquired by using a DeltaVision RT system. In **b**, **c**, and **e**, images are maximum-intensity projections, time is given in min:s; scale bars, 1 μm


We used a temperature-sensitive *cut7.24*^ts^ mutant in our spindle reassembly assay and set the final temperature to 37 °C to abrogate Cut7 activity. Similar temperatures do not disrupt spindle assembly and completion of mitosis in wild-type cells [40]. We estimate that Cut7 was inactivated within 3 min, given that it took about a minute to raise the temperature from 1 °C to 37 °C and the mutation response time was estimated to be 2 min [48]. We found that only 5 ± 5% (1 out of 19) spindles in *cut7.24*^ts^ cells reassembled at times longer than 3 min (Fig. 3a). In the remaining cells, the MTs extending from the two SPBs did not form an antiparallel bundle, often crossing each other in an X-shaped conformation (Fig. 3b; Additional file 3: Movie S2). The fraction of reassembled spindles was smaller than the fraction of reassembled spindles in wild type at times longer than 3 min (63 ± 6%, or 38 out of 60, calculated from data in Fig. 1d). To exclude the possibility that this difference is due to different distances between the SPBs, we took into account only the cells in which this distance was in the range 1–5 μm and found that 14 ± 8% (3 out of 21) spindles in the *cut7.24*^ts^ mutant reassembled, whereas in wild type this fraction was 58 ± 7% (31 out of 53). Additional comparisons are shown in Additional file 1: Figure S1 f. We conclude that Cut7 is important for the formation of an antiparallel bundle in the spindle reassembly assay.

**Additional file 3: Movie S2.** Movie of a *cut7.24*^ts^ cell expressing mCherry-tubulin (strain CF.391) at non-permissive temperature, in which the spindle did not reassemble. Images are maximum-intensity projections; time is given in min:s starting from time 0, scale bar, 1 μm. Time 0 is the time when the cold treatment ended and the temperature was raised to 37 °C. The movie corresponds to Fig. 3b. (MP4 3650 kb)


We also applied the spindle reassembly assay to the mutants lacking the proteins that have been shown to regulate spindle length in metaphase: kinesin-8 motor protein Klp5 [52, 53], kinesin-14 motors Pkl1 and Klp2 [43, 44], and the crosslinker Ase1 [50, 51]. We found that spindles were able to reassemble in the absence of these proteins (Additional file 1: Figure S1 g, S1 h). In all the studied mutants, the reassembly time increased with an increase in the distance between the SPBs, similarly to wild type, except in the *klp5*Δ mutant in which a similar correlation was not evident, possibly due to longer distances between the SPBs (Additional file 1: Figure S1i).

To compare *cut7.24*^ts^ cells with the other mutants and wild type, we analyzed only the cells that reassembled spindles at times longer than 3 min or did not reassemble. Whereas in wild type and all the mutants except *cut7.24*^ts^ more than 50% of the spindles reassembled, in *cut7.24*^ts^, this fraction was only 5% (Additional file 1: Figure S1j). Taken together, our experiments on a set of mutants suggest that Cut7 has a function in the transformation of oblique MT contacts into antiparallel bundles to reassemble the spindle.

### Cut7 is found at the contact site between microtubules during microtubule rotation into antiparallel alignment

To explore the localization of Cut7 and thus the potential sites where it may exert forces that align the MTs from the opposite SPBs into antiparallel configuration, we used cells expressing Cut7-3GFP as well as mCherry-tubulin and Sid4-mCherry in our assay (Fig. 3c; Additional file 1: Figure S2a-S2c; Additional file 4: Movie S3). During cold treatment, Cut7-3GFP showed a diffuse signal in the nucleus (Additional file 4: Movie S3). When the temperature was increased and MTs started to nucleate from the SPBs, Cut7 appeared close to the SPBs (*n* = 50 out of 50 cells; Fig. 3c, 0:10). We first analyzed the cells in which the SPBs were separated by more than 1 μm after the cold treatment (*n* = 19 out of 50 cells). We found that MTs pivoted around the SPBs and eventually formed a bundle connecting the SPBs (*n* = 15 out of 19 cells; Fig. 3c, 0:10–0:35), in agreement with our results shown in Fig. 1b. Interestingly, when MTs extending from the two SPBs established contact, Cut7 was found at the contact site (*n* = 8 out of 8 cells in which the initial MT contact was clearly visible; Fig. 3c, 0:25; in the remaining 7 cells, the initial MT contact site was unclear). Intensity profiles of the Cut7-3GFP signal along the contour length of MTs show that Cut7 appeared at the contact point and was present at this point during the process of MT alignment (Fig. 3d, 0:25–0:35). When the angle between the MTs changed from an oblique to the straight angle, Cut7 distribution changed from a spot to a broader distribution along the spindle, resulting in several Cut7 streaks along the spindle (*n* = 14 out of 15 cells; Additional file 4: Movie S3). Similar examples are shown in Additional file 1: Figure S2a and S2b.


**Additional file 4: Movie S3.** Spindle reassembly in a cell expressing Cut7-3GFP (green), mCherry-tubulin (magenta), and Sid4-mCherry (magenta; strain LW042). Images are maximum-intensity projections; time is given in min:s, scale bar, 1 μm. Time 0 is the time when the cold treatment ended, and the temperature was raised to 24 °C. The movie corresponds to Fig. 3c. (MP4 1460 kb)


In the cells in which the SPBs were separated by less than 1 μm after the cold treatment (*n* = 31 out of 50 cells), Cut7 was found at the SPBs upon temperature increase (n = 31 out of 31 cells). The spindles reassembled (*n* = 30 out of 31 cells), but it was not possible to observe the initial contact between the MTs extending from the opposite SPBs and the distribution of Cut7 at that time due to the short distance between the SPBs (Additional file 1: Figure S2c). Note that the kinetics of spindle reassembly in the cells expressing Cut7-3GFP (Additional file 1: Figure S2d) was similar to that in wild-type cells shown in Fig. 1d, suggesting that labeling of Cut7 did not perturb this process.

Based on our experiments in which Cut7 and MTs were visualized, we conclude that Cut7 near the SPBs cannot contribute to the alignment of MTs extending from the opposite SPBs and interacting at an oblique angle, because all MTs extend from the same SPB in that region. We speculate that Cut7 found at the site of MT interaction may exert forces that align the MTs into antiparallel configuration.

Finally, kinetochores may have a role in MT alignment, because interactions between kinetochores and microtubules are important for spindle structure [54]. To investigate this possibility, we followed the kinetochores during spindle reassembly and found that MT alignment occurs without kinetochores being present close to the MT contact point (Fig. 3e; Additional file 1: Figure S2e and S2f). We also found that spindle reassembly time was similar in cells that have a free kinetochore in the nucleoplasm and in those in which all the kinetochores were at the SPBs (12 ± 3 min, *n* = 19, and 11 ± 3 min, *n* = 24, respectively; here we used cells with *d*_SPB_ > 1.5 μm to compare cells with similar *d*_SPB_ in both groups, see Additional file 1: Figure S2f). These results suggest that kinetochores do not significantly influence the formation of antiparallel MT bundles.

### Theoretical model

**Fig. 4:**
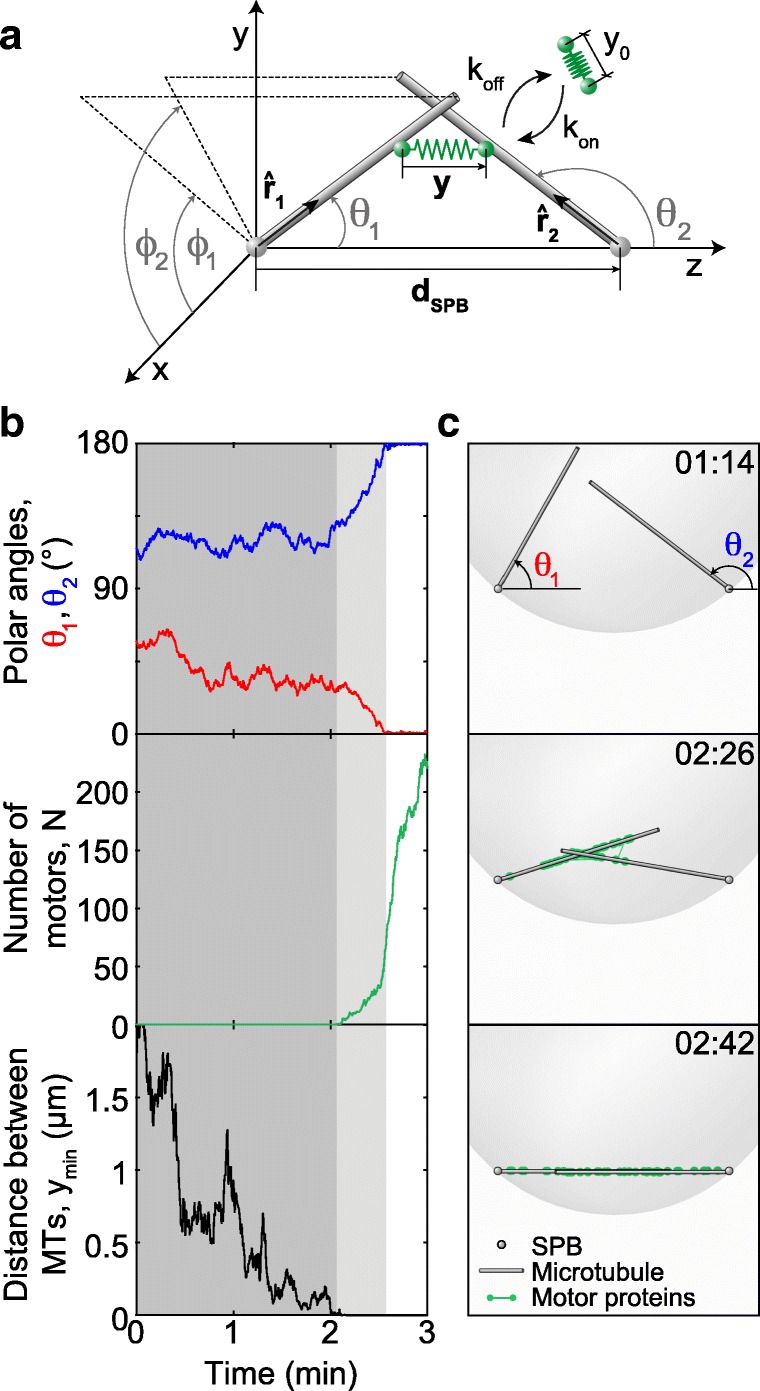
Theoretical model and solutions for MT dynamics. **a** Scheme of the model. Each MT (gray rod) is freely joint to its respective SPB (gray sphere). Orientations of two MTs are represented with unit vectors ${\hat{\mathbf{r}}}_1$ and ${\hat{\mathbf{r}}}_2$ respectively, while the SPBs are at fixed points separated by the distance ***d***_SPB_. Motor proteins (green springs with rest length *y*_0_) can attach to and detach from MTs with rates *k*_on_ and *k*_off_, respectively, and when attached, their elongation is ***y***. In the Cartesian coordinates, the SPBs are at points (0, 0, 0) and (0, 0, *d*_SPB_). The MT orientations are described by the polar angles *θ*_1_ and *θ*_2_ and by the azimuthal angles *φ*_1_ and *φ*_2_ for the first and the second MT, respectively. **b** A sample path representing a bundling event. Top, polar angles denoted with a blue and a red line for the first and second MT, respectively. In the antiparallel configuration, *θ*_1_ = 0°, *θ*_2_ = 180° (see **a** for parametrization). Middle, number of attached motors. Bottom, distance between the two closest points on the MTs. Shaded regions: the search (dark gray) and the aligning phase (light gray); white region represents the bundled state. Simulation is performed with parameter values *R*_1,2_ = 1.5 μm, *d*_SPB_ = 2 μm, *n*_MT_ = 2, and other values from Table 1. **c** Illustrations of search (top), aligning (middle), and bundled state (bottom) of MTs (gray rods). To illustrate the motor distribution, the position of each motor (green) is randomly generated using a normal distribution around their mean position with the steady state variance, which is calculated from the MT orientations. The small gray spheres represent the SPBs and the large translucent gray sphere represents the nuclear envelope. The images are taken from Additional file 5: Movie S4, which is produced using the same data as in **b**. Time is given in min:s

To explore how the MTs, which extend in arbitrary directions, become aligned into an antiparallel bundle connecting the spindle poles, we introduce a simple physical model (Fig. 4a and Methods). The central idea of our theoretical approach is that MTs perform rotational movement around the spindle pole, allowing them to explore the space as they search for the MTs extending from the opposite pole and to establish a configuration required for spindle assembly. In our model, two types of forces drive the rotational movement of MTs: forces generated by motor proteins and thermal forces. The forces generated by motors appear when MTs get into close proximity allowing the motors to attach in this region and thus crosslink the MTs. In our model, motors attach simultaneously with both ends to two MTs extending from the opposite spindle poles (Fig. 4a). A motor is described as an elastic spring, whose two ends can move along two MTs. The motors move towards the MT minus end, which is at the spindle pole, generating a directed force on the MTs that rotates them towards the pole-pole axis. A motor is considered as a force generator, whose velocity decreases under load. In contrast to motor-generated forces, thermal forces are random and always present irrespective of the distance between the MTs. To keep the model simple, we consider straight MTs of a constant length extending from each spindle pole. MTs are pinned at one end at the nuclear envelope of a spherical shape. We use this model to calculate the dynamics of antiparallel bundle formation.

We solved the model numerically to obtain the time course for MT orientations and the number of attached motors. MT orientations are parametrized by angular coordinates, the polar and the azimuthal angle (Fig. 4a). For parameters given in Table 1 and discussed in the Methods section, solutions of the model show that MTs initially preform random angular movement (Fig. 4b, top). This movement of MTs is predominantly driven by thermal forces, as there are no motors attached to them (Fig. 4b, middle) because the MTs are not yet in contact (Fig. 4b, bottom). Our calculations show that this random movement ends when the MTs come close enough to each other so that motors can attach (Fig. 4b, bottom). Subsequently, polar angles change in a directed manner towards the antiparallel configuration (light gray region in Fig. 4b, top). This directed movement is the result of the accumulation of motors that generate forces (Fig. 4b, middle). The movement stops when the polar angles approach 0° and 180° for the first and the second MT, respectively (Fig. 4b, top), thereby forming a stable antiparallel bundle. This directed movement of MTs and the accompanying accumulation of motors correspond to the experimental observations that characterize the alignment step (Figs. 2 and 3). In the model, the motors accumulate most rapidly after the MTs have become aligned (white region in Fig. 4b, middle), which is not observed in experiments (Fig. 3c, d). This difference may be due to a larger MT overlap and a larger pool of motors in the model compared to experiments. The entire time course of the MTs and the behavior of motors calculated from the model and corresponding to Fig. 4b is illustrated in Additional file 5: Movie S4, and the still frames from the animation representing the search, aligning and bundled state are shown in Fig. 4c, top, middle, and bottom, respectively.

**Table 1:** Parameters used in the model

		Value	Source
Motor parameters			
*v*_0_	Motor velocity	−0.01 μm/s	Estimated from contour velocity
*D*_c_	Motor velocity fluctuation	5 × 10^−4^μm^2^/s	[55]
*f*_0_	Motor stall force	−1.5 pN	[56]
*k*_on_ *c*_nuc_	Motor concentration parameter	0.5 s^−1^	Estimated
*k*_off_	Motor detachment rate	0.1 s^−1^	Estimated from [19]
*k*	Motor stiffness	300 pN/μm	[57]
*y*_0_	Motor rest length	53 nm	[58]
Microtubule parameters			
*n*_MT_	Number of MTs in a nucleus	10	Measured here
〈*R*_1,2_〉	Expected MT length	0.8 μm	[29, 59]
*D*_1,2_	MT diffusion constant	$0.003/{R}_{1,2}^3$	[29]
Other parameters			
*d*_SPB_	Distance between SPBs	0.5 − 2.5 μm	Measured here
*R*_C_	Nuclear envelope radius	1.5 μm	[29, 60]

The choice of parameters values is described in Methods


**Additional file 5: Movie S4.** Animation of the MT bundling process calculated by using the model. Data for the orientations of MTs (gray rods) as well as the distribution of the motors (green) are taken from the same simulation run used in Fig. 4b. To illustrate the motor distribution, the position of each motor is randomly generated using a normal distribution around their mean position with the steady state variance, which is calculated from the MT orientations. The small gray spheres represent the SPBs and the large translucent gray sphere represents the nuclear envelope. The movie corresponds to Fig. 4c. (MP4 1540 kb)


### The model predicts that bundle formation is faster for small distances between the SPBs, large MT number, and fast MT diffusion

**Fig. 5:**
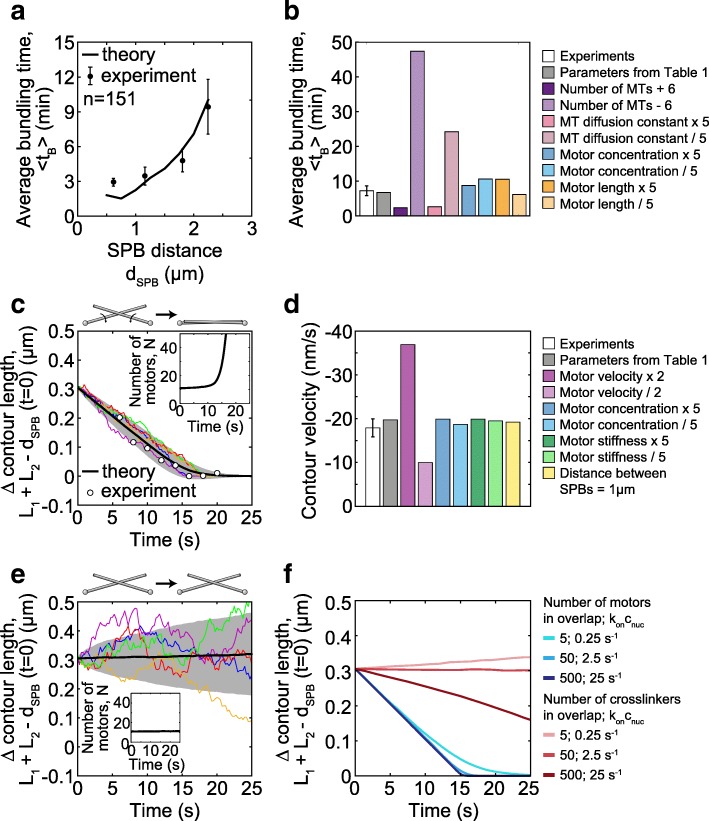
Bundling time and contour length. **a** Average bundling times in theory (black line) and experiments (circles with error bars) measured as spindle reassembly time for *d*_SPB_ < 2.5 μm (*n* = 109, strain KI061; *n* = 42, strain LW042). The average bundling time, 〈*t*_B_〉 = *t*_tot_/*n*_B_, where *t*_tot_ is the total time the MTs were observed or simulated and *n*_B_ is the number of bundling events in that time. **b** Average bundling times in experiments, for 1.5 μm < *d*_SPB_ < 2.5 μm (white bar, data from **a**), and in theory for varied parameters (colored bars, *d*_SPB_ = 2 μm). **c** Contour length difference, calculated as *L*_1_ + *L*_2_ − *d*_SPB_, (main graph) and the number of motors (inset) as a function of time. In the simulations, MTs start from a symmetric configuration and *α* = 120°, *y*_min_ = 0 μm (see Methods). Average value (black line), standard deviation (shaded area), 5 sample paths for simulations (colored lines) for *d*_SPB_ = 2 μm, *R*_1,2_ = 2 μm, and *n*_MT_ = 2. Mean experimental values (white dots) from Fig. 2d (from cells where the motors were not tagged in order to avoid potential effects of tagging on motor velocity), with times shifted by 16 s. **d** Contour velocity in experiments (white bar; data from Fig. 2d) and in theory for varied parameters (colored bars); *R*_1,2_ = 2 μm, *d*_SPB_ = 2 μm, except for the last bar *R*_1,2_ = 1 μm, *d*_SPB_ = 1 μm. **e** Contour length difference (main graph) and the number of passive crosslinkers (inset) as a function of time, with same color code as in **c**. Parameters for passive crosslinkers: diffusion constant *D*_c_ = 0.05 μm^2^/s [61], and the rest length *y*_0_ = 40 nm [62]. **f** Average contour length difference from theory for passive crosslinkers (red) and motors (blue) as a function of time, for parameters given in the legend and in **e**. In panels **d**–**f**, initial geometry is the same as in **c**. Parameters in all panels are from Table 1 unless stated otherwise. Error bars, s.e.m

To provide a quantitative measure that can be compared with experiments, we calculate the average bundling time, defined as the time required for MTs to form an antiparallel bundle, which takes into account both steps in bundle formation, i.e., search and alignment (Methods). We found that the average bundling time is roughly 1–10 min for parameters in Table 1 and random initial conditions (Fig. 5a). The average bundling time increases as the SPB distance increases (Fig. 5a). To compare these results with experiments, we calculated the average spindle reassembly time for different distances between the SPBs in wild-type cells and found that our model reproduces the experimental measurements (Fig. 5a). There is a discrepancy at small SPB distances, possibly due to the smaller MT numbers at the onset of MT growth (Fig. 1e), which is the time window relevant for reassembly in this case. We conclude that the agreement between the model and experiments supports the hypotheses used to build our model.

We further explored the predictions of the model by varying the parameters of the model and calculating the resulting average bundling time (Fig. 5b). We found that an increase in MT number by a factor of 1.6 accelerates bundle formation by a factor of 3, whereas a decrease in MT number slows down this process. Similarly, MT diffusion affects bundle formation, though to a smaller extent (Fig. 5b). On the other hand, the concentration of motors and their rest length have a minor contribution even when changed by a factor of 5 (Fig. 5b). The concentration of motors is not a critical parameter within the tested range, as long as a few motors can bind to the MT contact point, which are sufficient to drive the formation of an antiparallel bundle. Thus, bundling time depends mainly on MT number and diffusion.

### Minus-end-directed motors align the MTs into an antiparallel bundle at a constant velocity

In our experiments, we found that the contour length of MTs decreased during their rotation into antiparallel alignment. To help us interpret these results, we used the model to explore the change in MT contour length. Here we start our calculations from the configuration in which the MTs are in contact (Fig. 5c is for a symmetric and Additional file 1: Figure S3a for an asymmetric initial configuration). We find that the contour length difference decreases towards zero and remains constant afterwards (Fig. 5c). This process is driven by motors, which initially accumulate slowly, whereas the accumulation accelerates when the MTs approach the aligned configuration (Fig. 5c, inset). The contour length decreases linearly at a velocity close to 2*v*_0_, twofold the velocity of motors along each MT. The value of the parameter *v*_0_ was chosen to reproduce the experimentally measured contour velocity (Table 1 and Methods). To explore the predictions of the model, we varied the parameters and found that the contour velocity is proportional to the motor velocity, whereas the concentration of motors, their stiffness, and the SPB distance have a minor contribution within the tested range (Fig. 5d; Additional file 1: Figure S3b; other parameters are investigated in Additional file 1: Figure S3c). Note that our model does not include crowding of motors and the accompanying changes of motor directionality [20].

Interestingly, the prediction that the contour velocity does not depend on the SPB distance implies that this velocity is robust to changes in the geometry of the system. To test this prediction, we divided the cells into two groups, those with the SPB distance smaller or larger than 1.85 μm. Indeed, we found that the contour velocity was not different between these groups (− 18 ± 5 nm/s and − 24 ± 6 nm/s for the cells with SPB distance of 1.69 ± 0.03 μm and 2.3 ± 0.2 μm, respectively; *n* = 14; *p* = 0.5 from a t-test for velocities). Taken together, our results suggest that the minus-end-directed motors align the MTs into an antiparallel bundle and allow us to identify their velocity.

Our model together with experiments shows that the search times are several-fold longer than the duration of MT aligning. Thus, the average bundling time, which includes both search and aligning, describes predominantly the time scale of the search process, which is on the order of minutes. The change of contour length captures only the time scale of the aligning step, which is on the order of seconds. Our model, which is based on the known properties of MTs and motors, explains the process of antiparallel bundle formation covering different time scales.

An alternative scenario is that motor activity is not required for antiparallel bundle formation, but passive crosslinkers may drive this process [34, 35]. In order to investigate this possibility, we use our model to describe the interactions between MTs mediated by passive crosslinkers (Methods and Fig. 5e). In contrast to the case with motors, we find that with passive crosslinkers, the contour length is almost constant over time and MT alignment does not occur on relevant time scales (Fig. 5e), even though crosslinkers remain attached to the MTs (inset in Fig. 5e). The experimentally observed alignment is not reproduced even for a two orders of magnitude higher concentration of crosslinkers, whereas the alignment driven by motors is in agreement with experiments irrespective of motor concentration (Fig. 5f). Yet, passive crosslinkers can drive MT alignment on a relevant time scale if the distance between the SPBs is small and the number of crosslinkers is high (Additional file 1: Figure S3d). Thus, the model with passive crosslinkers instead of motors cannot reproduce MT alignment observed in our experiments.

## Discussion

### Microtubule pivoting around the spindle pole facilitates their encounter

Our results based on live-cell imaging provide a direct observation of interpolar bundle formation in vivo, which is a missing piece of information critical for the understanding of spindle assembly. By using a spindle reassembly assay, we found that the formation of an antiparallel bundle occurs in two steps, search and aligning (Fig. 1c). During the search step, MTs extending from the opposite SPBs rotate around the SPB, which helps the MTs to find each other. MT rotation during the search step is passive angular diffusion, which is thermally driven and does not require ATP [29]. Previous computer simulations of spindle assembly in fission yeast indicate that a decreased MT rotation results in fewer MTs in the bundle connecting the two SPBs and shorter spindles [32]. Thus, previous work and our model together with experiments show that rotation of MTs is required for the process in which they search for each other to form an antiparallel bundle.

MT rotation has been observed before in fission yeast during mitosis and meiosis [29, 59], in budding yeast [63], and in *Drosophila* S2 cells [64]. In general, pivoting helps the MTs as they search for targets such as kinetochores [29, 59, 65], cortical anchors in vivo [63] and in vitro [66], or other MTs [30, 64]. This motion allows MTs to swipe through space, which increases the explored volume and makes the search process more efficient [2, 67].

### Dynamics of the microtubule contour length reveals that minus-end-directed motors align the microtubules into antiparallel bundles

During the second step of bundle formation, MTs extending from the opposite SPBs rotate towards the pole-to-pole axis to form an antiparallel configuration. This rotation occurs after the MTs have established contact at an arbitrary angle. Whereas MT rotation is random before the contact, it becomes directed as they pivot towards the antiparallel alignment after the contact. The idea that MTs rotate into antiparallel alignment during spindle assembly was introduced in a previous study [31]. Here, we were able to observe and quantify this directed rotation by using a spindle reassembly assay to increase the distance between the spindle poles,.

During early mitosis in unperturbed cells, while the duplicated SPBs are still connected by a bridge, electron micrographs have shown that the MTs from each SPB interdigitate at sharp angles, whereas antiparallel bundles are not yet visible [28]. Thus we propose that MTs rotate to become aligned and form an antiparallel interpolar bundle also during normal spindle assembly. Even though the angles at which the MTs intersect are larger in our assay than in unperturbed cells, the mechanism of rotation may be similar in both cases because motors that link two MTs and move towards their minus ends can align the MTs into an antiparallel bundle irrespective of the initial angle between the MTs. Alternatively, in unperturbed cells the interpolar bundles required for spindle assembly could form through capture of MTs emanating from one SPB by kinesin-5 motors clustered at the opposite SPB, which subsequently induce antiparallel sliding of the MTs [68]. In higher eukaryotic cells, the majority of interpolar bundles form when the centrosomes are apart [69], which is similar to our reassembly assay and thus our results on search and alignment are likely relevant for these systems.

During MT alignment, MTs slide sideways along each other like a skater on a handrail. We introduce the contour length of MTs as a measure of activity of motors that drive MT sliding. This measure provides information about motor directionality and velocity. Thus, the approach developed in this work may be used to study the activity of any motor of interest or their combinations in a mitotic context that has not been studied so far.

Our finding that the contour length of MTs decreases during their rotation, together with our theory, implies that the motors accumulated at the contact site walk towards the minus ends. Our measurement that the contour length decreases at a velocity of roughly 20 nm/s indicates that the motors walk at this velocity. This reasoning holds for fission yeast spindles where poleward flux is absent [41]. In the case of a tetrameric motor, the motor walks along each MT at a half of that velocity.

The velocity measured here is similar to the velocity of the minus-end-directed motility of kinesin-5 and kinesin-14 motors, but smaller than dynein velocity, in yeasts. In gliding assays in vitro, the kinesin-5 Cut7 from fission yeast moves at a velocity of 30 nm/s [23], and Cin8 from budding yeast at 30–50 nm/s [19]. The kinesin-14 Pkl1 from fission yeast moves at a velocity of 33 nm/s in a gliding assay [70], while the truncated Kar3 from budding yeast moves at 20 nm/s in a gliding assay [71] and the full-length Kar3-Cik1 at 45 nm/s in a stepping assay [31]. Finally, cortically anchored dynein in fission yeast moves the nucleus at a velocity of 100 nm/s [72], and single dyneins move at 140 nm/s along the MT [73], whereas full-length purified budding yeast dynein glides MTs in vitro at 90 nm/s [74].

### Molecular players involved in resolving oblique microtubule contacts

We propose that Cut7, a kinesin-5 family member, plays a role in MT rotation into antiparallel alignment, based on the literature and our experiments on candidate motor proteins and a non-motor crosslinker. Previous works have shown that Cut7 is essential for spindle formation [13], unlike the other 8 kinesins of *S. pombe* [43–48], dynein [49] and the non-motor crosslinker Ase1/PRC1 [50, 51]. The key role of Cut7 in spindle formation was revealed by inactivation of Cut7 in temperature-sensitive mutants, which resulted in cells with monopolar spindles [13]. Similarly, Cut7 inactivation during metaphase leads to spindle collapse into an aster pattern [48]. Electron micrographs of monopolar spindles produced by Cut7 inactivation showed that MTs extending from the two SPBs are roughly parallel, suggesting that Cut7 is required for MT interdigitation [75]. Our experiments showing accumulation of Cut7 at the site of initial MT contact, together with low efficiency of spindle reassembly in a temperature-sensitive *cut7.24*^ts^ mutant, are consistent with a role of Cut7 in the formation of antiparallel MT bundles starting from oblique MT interactions. Importantly, our data provide experimental evidence for minus-end-directed motility of Cut7 in vivo and the biological role of this directionality. It would be interesting to apply the spindle reassembly assay to *cut7* double mutants, to cells with targeted *cut7* mutations, and to those expressing Cut7 forms of different directionalities, in order to explore the role of specific interactions and potential additional mechanisms.

Other motors, such as the minus-end-directed kinesin-14 motors Pkl1 and Klp2 [43, 44] and dynein [49] might contribute to MT rotation into antiparallel alignment. Yet, we found that spindles were able to reassemble in cells lacking Pkl1 or Klp2, which is consistent with previous observations that spindles can reassemble in cells lacking Pkl1, Klp2 or dynein after MT depolymerization in kinetochore capture assays [39, 76]. Additionally, cells lacking any of these three motors or even all three of them are able to form spindles under normal conditions [44, 49]. Thus, Pkl1, Klp2 and dynein are not crucial for the resolution of oblique MT contacts during spindle assembly.

Finally, it is possible that MT rotation into antiparallel alignment occurs without motor activity. Our theoretical results show that MT alignment can be driven by passive crosslinkers for small distances between the spindle poles, in agreement with previous works [33–35]. However, for spindle pole distances relevant for our experiments, our theory together with our measurements of contour length suggests that the action of minus-end-directed motors is required for MT alignment. Our experiments on Cut7 together with the previously observed minus-end-directed motility of Cut7 [23] suggest a role of Cut7 in this process.

### Change in the direction of forces after microtubule alignment

While our model together with experiments indicates that motors walk towards the MT minus end to align MTs, this minus-end-directed motility is expected to shorten the bundle after it is formed. Yet, we observed that the spindle remained at a constant length or elongated slowly upon reassembly, which is indicative of forces acting in the opposite direction. Thus, our work suggests that there is a switch in the direction of forces during spindle reassembly, and the underlying mechanisms are currently unknown. It may be that forces generated by other molecular players, which push the spindle poles apart, start to dominate over the minus-end-directed motors. These forces may be generated by plus-end-directed motors such as kinesin-6/Klp9 [48], or by pushing forces generated by the interaction of growing MT plus ends with the opposite SPB [33, 34]. Moreover, the motors that align the MTs into the antiparallel bundle may change their direction of motion after the bundle is formed. Crowding on the MT has been suggested to convert the Cut7 motor from minus-end-directed to plus-end-directed stepping [20]. Similarly, single Cin8 motors from budding yeast were shown to move towards the minus end on individual MTs, but they switch to plus-end-directed motility when working in a group of motors on antiparallel MTs [19, 21]. Exciting new experimental and theoretical investigations await in this field to reveal how the regulation of motor protein activity and MT dynamics govern spindle formation and function.

## Methods

### Strains and sample preparation

The strains (Additional file 1: Table S1) were obtained by crossing, followed by random spore analysis [77]. The cells were grown on Yeast Extract with supplements (YES) medium agar plates at 25 °C [77]. A loopfull of cells was further cultured in liquid YES medium in a shaking incubator (ISF-1-W, Kuehner Shaker, Birsfelden, Switzerland) at 25 °C for 2–3 h. For the strains CF.391, I1_2_10, KI013, and LW042, 3 mM hydroxyurea (Sigma-Aldrich) was added to liquid YES medium in order to obtain lengthy cells with normal MT dynamics [78, 79] and with lengthy spindles, the cells were kept for 11–14 h in the shaking incubator at 25 °C, and subsequently the liquid culture was diluted with liquid YES medium at a ratio 1:3. The wall of a 35 mm (No1.5) culture dish (MatTek Corporation, Ashland, MA, USA) was cut to 2 mm height. The original coverslip was removed from the dish bottom and the remaining dish was soaked in 70% ethanol overnight. Cover slips (Corning 22 mm × 22 mm, Sigma-Aldrich) were washed in 2-propanol and attached to the pre-washed culture dish with transparent nail polish. The dish was coated with lectin (L2380, Sigma-Aldrich, St Louis, MO, USA) 30 min prior to usage. 200 μl of liquid culture was placed on the pretreated culture dish for 25 min for sedimentation. The cells were washed 3 times with 200 μl of YES medium. The dish was closed with a cover slip (Corning, Inc.) to prevent the sample from drying out.

### Microtubule depolymerization by cold shock

To quickly depolymerize metaphase spindles, a thermoelectric device based on a Peltier element was designed and tested with an independent type K thermocouple (Omega Engineering, Deckenpfronn, Germany) and a Fluke 50 Serie II Thermometer (Fluke Corporation, Everett, WA, USA). Prepared samples were loaded onto the microscopy stand and a pre-cooled thermoregulation at 15 °C, to slow down mitosis and thus facilitate our search for a field of view with a high number of cells in metaphase (Additional file 1: Figure S1a). Following image acquisition, the temperature was set to 0 °C. Once set to 0 °C, the temperature dropped to 1 °C within 60 s inside the sample and was maintained typically for 15 min. To ensure a constant temperature, the objective was lowered to at least 2 cm away from the sample dish. Subsequently, the objective was returned to the initial position, the same field of view was placed into focus, and image acquisition was initiated. Within 20 s of acquisition, the temperature was set to 24 °C. The temperature inside the sample reached this value within 30 s. Because of the change in temperature, the sample was manually refocused during the acquisition. Once the temperature in the sample was stabilized at 24 °C, the focus remained constant. In experiments with *cut7.24*^ts^ cells, the final temperature was set to 37 °C instead of 24 °C to inactivate Cut7. Live-cell imaging was performed for 10 min.

### Time-lapse live cell imaging

Live images were taken using an Andor Revolution Spinning Disk System (Andor Technology plc., Belfast, United Kingdom), consisting of a Yokogawa CSU10 spinning disk scan head (Yokogawa Electric Corporation, Tokyo, Japan) with a 405/488/568/647 Yokogawa dichroic beamsplitter (Semrock, Inc., Rochester, NY, USA). The scan head was connected to an Olympus IX71 inverted microscope (Olympus, Tokyo, Japan) equipped with a fast piezo objective z-positioner (PIFOC, Physik Instrumente GmbH & Co. K.G., Karlsruhe, Germany) and an Olympus UPlanSApo 100x/1.4 NA oil objective (Olympus, Tokyo, Japan). For cells expressing GFP and tdTomato, we performed sequential imaging (2 s time interval between each image pair) or simultaneous acquisition (1 s time interval between images) using a DualView image-splitter (Optical Insights, Photometrics, Tucson, AZ, USA). Cells expressing only GFP (Mal3-GFP) were imaged with 250 ms time interval. Exposure time was 20 ms. For excitation, a Sapphire 488 nm solid-state laser (75 mW; Coherent, Inc., Santa Clara, CA, USA) and a Jive 561 nm solid-state laser (75 mW; Cobolt, Stockholm, Sweden) were used for GFP and tdTomato, respectively. Laser intensity was controlled using the acousto-optic tunable filter inside the Andor Revolution Laser Combiner (ALC, Andor Technology plc., Belfast, UK). For sequential imaging, emission wavelength was selected using respective emission filters BL 525/30 (Semrock, Inc., Rochester, NY, USA) and ET 605/70 (Chroma, Bellows Falls, VT, USA) mounted in a fast, motorized filter wheel (Lambda-10B, Sutter Instrument Company, Novato, CA, USA). The microscope was equipped with an iXon EM+ DU-897 BV back-illuminated Electron Multiplying Charge Coupled Device (EMCCD, Andor Technology plc., Belfast, UK), cooled to − 80 °C, electron multiplication gain 300. The resulting xy-pixel size in the images was 168 nm. The system was controlled by Andor iQ software version 2.9 (Andor Technology plc., Belfast, UK). For short-term acquisitions (10–20 s), sequential time-lapse z-stacks (2-s time interval between each image pair) of 13 optical sections with 0.5-μm z-spacing was performed using a DualView image-splitter (Optical Insights, Photometrics). For main acquisitions (10-min), time-lapse z-stacks of 13 optical sections with 0.5-μm z-spacing were taken every 2 s with exposure times of 0.06 and 0.08 s. In the case of main acquisitions (10-min) of strain LW042, sequential time-lapse z-stacks (5-s time interval between each image pair) of 13 optical sections with 0.5-μm z-spacing was performed, with exposure times of 0.08 and 0.1 s for GFP and mCherry, respectively.

### Theoretical model

#### Orientations of the MTs

We model the MTs as two thin, rigid rods of fixed length *R*_1_ and *R*_2_ (here and in the rest of this text, indices 1 and 2 represent the first and the second MT, respectively), each with one end freely joint (pinned, but not clamped) at the respective SPB. Their orientations are represented by unit vectors ${\hat{\mathbf{r}}}_{1,2}$ (Fig. 4a), and the SPBs are positioned at the origin and at ${\mathbf{d}}_{\mathrm{SPB}}={d}_{\mathrm{SPB}}\hat{\mathbf{z}}$, with $\hat{\mathbf{z}}$ being the unit vector in the direction of the Cartesian z-axis. The MTs pivot around their respective SPB with the angular velocities **ω**_1,2_. The orientations of MTs change in time, *t*, as

1 $$ \frac{\mathrm{d}{\hat{\mathbf{r}}}_{1,2}}{\mathrm{d}t}={\boldsymbol{\upomega}}_{1,2}\times {\hat{\mathbf{r}}}_{1,2}. $$

In the overdamped limit, the angular friction experienced by the MTs is balanced by the total torque,

2 $$ {\gamma}_{1,2}{\boldsymbol{\upomega}}_{1,2}={\mathbf{T}}_{1,2}, $$

where *γ*_1,2_ is the angular friction coefficient of the MTs. The total torque consists of two contributions, ${\mathbf{T}}_{1,2}={\boldsymbol{\uptau}}_{1,2}+{\sigma}_{1,2}\left[{\hat{\mathbf{r}}}_{1,2}\times {\boldsymbol{\upeta}}_{1,2}(t)\right]$, where the first term is the deterministic torque, **τ**_1,2_, caused by the forces exerted by the crosslinking proteins attached to the MTs and the second term is the stochastic term describing the noise. In our model, the noise is thermal, so its intensity is calculated from the equipartition theorem as ${\sigma}_{1,2}=\sqrt{2{k}_{\mathrm{B}}T{\gamma}_{1,2}}$, with *k*_B_*T* being the Boltzmann constant multiplied by the temperature. The 3-dimensional random vector **η**_1,2_ has components that are normally distributed with zero mean and unit variance. The noise is uncorrelated in time and its components are independent, 〈*η*_*i*_(*t*), *η*_*j*_(*t*^′^)〉 = *δ*(*t* − *t*^′^)*δ*_*ij*_, with *δ*(*t* − *t*^′^) being the Dirac delta function and *δ*_*ij*_ is the Kronecker delta function. Using these definitions and eq. (2), we obtain the equations for the angular velocities of the MTs,

3 $$ {\boldsymbol{\upomega}}_{1,2}={D}_{1,2}\frac{{\boldsymbol{\uptau}}_{1,2}}{k_BT}+\sqrt{2{D}_{1,2}}\left[{\hat{\mathbf{r}}}_{1,2}\times {\boldsymbol{\upeta}}_{1,2}(t)\right], $$

where *D*_1,2_ = *k*_B_*T*/*γ*_1,2_ denotes the angular diffusion coefficient of the MTs.

#### Forces, torques and movement of crosslinking proteins

The torques in Eq. (3) depend on the distributions of the crosslinking proteins which are attached at a given time, ${\boldsymbol{\uptau}}_{1,2}={\sum}_{i=1}^N{\mathbf{r}}_{1,2;i}\times {\mathbf{f}}_{1,2;i}$. The indices *i* = {1, .., *N*}, refer to crosslinking proteins attached to both MTs.

A crosslinking protein that is attached to the MTs is modeled as a Hookean spring with the rest length *y*_0_, and ends attached at the positions ${\mathbf{r}}_{1,2;i}={r}_{1,2;i}{\hat{\mathbf{r}}}_{1,2}$ from the respective SPBs, where *r*_1,2;*i*_ is the position along the MTs. The vector describing the elongation of the spring is **y**_*i*_ = **r**_2;*i*_ + **d**_SPB_ − **r**_1;*i*_, and the elastic force exerted by the spring reads

4 $$ {\mathbf{f}}_{1,2;i}=\pm k{\mathbf{y}}_i\left(1-\frac{y_0}{\left|{\boldsymbol{y}}_i\right|}\right), $$

where *k* is the spring stiffness.

The model introduced so far applies to any type of crosslinking proteins, including motor proteins and passive crosslinkers. However, the velocity of a crosslinking protein along the MTs depends on whether its movement is driven by an active process, as in the case of motor proteins, or it is thermal, as in the case of passive crosslinkers. If the crosslinking protein behaves as a motor protein, its velocity along MTs is given by

5 $$ {v}_{1,2;i}(t)={v}_0\ \left[1-\frac{{\mathbf{f}}_{1,2;i}\bullet {\hat{\mathbf{r}}}_{1,2;i}}{{\mathrm{f}}_0}\ \right]+\sqrt{2{D}_{\mathrm{c}}}{\eta}_{1,2;i}(t), $$

where the first, deterministic term is the motor velocity described by a linear force-velocity relationship and the second, stochastic term describes velocity fluctuations. Velocity of a motor head at zero load and the stall force are denoted *v*_0_, and *f*_0_, respectively, while *D*_c_ is the variance of the velocity fluctuations. On the other hand, if the crosslinking protein behaves as a passive crosslinker, its velocity along the MTs is given by

6 $$ {v}_{1,2;i}(t)=-\frac{D_{\mathrm{c}}}{k_{\mathrm{B}}T}{\mathbf{f}}_{1,2;i}\bullet {\hat{\mathbf{r}}}_{1,2;i}+\sqrt{2{D}_{\mathrm{c}}}{\eta}_{1,2;i}(t). $$

As expected from the fluctuation-dissipation theorem, both the friction coefficient, *D*_c_/*k*_B_*T* in the first term and the noise intensity $\sqrt{2{D}_{\mathrm{c}}}$ in the second term depend on the diffusion constant of crosslinkers along MTs, denoted *D*_c_. We will refer to the Eqs.(5) and (6) as the equations of motion for motors and passive crosslinkers, respectively.

#### Crosslinking protein attachment and detachment

Finally, the crosslinking proteins can attach to and detach from the MTs, so their total number *N*(*t*) changes in time. In every small time interval *∆t*, a new crosslinking protein can attach to both MTs with probability *k*_on_*N*_0_*∆t*, where *N*_0_(**y**) is the effective number of crosslinking proteins in the nucleoplasm that takes into account the energy of their stretching (see Eq. (15) below). Alternatively, an already attached crosslinking protein can detach with the probability *k*_off_*N∆t*. The constants *k*_on_ and *k*_off_ are the attachment and detachment rates, respectively. It is important to note that the crosslinking protein indices *i* = {1, .., *N*} have to change to account for the attachment or detachment events every time they occur.

### Solutions of the model

To obtain the time course of the MT orientations, we parameterize the orientation of the MT given by the unit vector by ${\hat{\mathbf{r}}}_{1,2}\left({\theta}_{1,2},{\phi}_{1,2}\right)=\left(\sin {\theta}_{1,2}\cos {\phi}_{1,2},\sin {\theta}_{1,2}\sin {\phi}_{1,2},\cos {\theta}_{1,2}\right)$, where *θ*_1,2_ and *ϕ*_1,2_ denote the polar and azimuthal angle, respectively. In this parameterization, equation (3) yields the equations of motion for the angles [30],

7 $$ \frac{\mathrm{d}}{\mathrm{d}t}\left[\begin{array}{c}{\theta}_{1,2}\\ {}{\phi}_{1,2}\end{array}\right]=\frac{D}{k_{\mathrm{B}}T}\left(\sum \limits_{i=1}^N{r}_{1,2;i}{\mathbf{F}}_{1,2;i}\right)\circ {\mathbf{a}}_{1,2}+D\left[\begin{array}{c}\cot {\theta}_{1,2}\\ {}0\end{array}\right]+\sqrt{2{D}_{1,2}}\left[\begin{array}{c}{\eta}_{\uptheta; 1,2}\\ {}\csc {\theta}_{1,2}{\eta}_{\phi; 1,2}\end{array}\right], $$

where ∘ represents the Haddamard (element-wise) product. For compactness, we introduced a term with dependence on positions of the crosslinking proteins that has dimension of force,

8 $$ {\mathbf{F}}_{1,2;i}\equiv k\left[\begin{array}{c}\left({r}_{2,1;i}-{L}_{2,1}\right)\\ {}{r}_{2,1;i}\end{array}\right]\left(1-\frac{y_0}{y_i}\right), $$

And a term that depends only on the angles,

9 $$ {\mathbf{a}}_{1,2}\equiv \left[\begin{array}{c}\left(\sin {\theta}_{2,1}\cos {\theta}_{1,2}\cos \left({\phi}_{1,2}-{\phi}_{2,1}\right)-\cos {\theta}_{2,1}\sin {\theta}_{1,2}\right)\\ {}\sin {\theta}_2\sin \left({\phi}_{2,1}-{\phi}_{1,2}\right)\end{array}\right]. $$

Here, the coordinates on the MTs where they are closest to each other are denoted *L*_2,1_. Because of the fact that the summation in equation (7) changes after attachment or detachment events, it is useful to rewrite it in terms of the average values,

10 $$ \sum \limits_{i=1}^N{r}_{1,2;i}{\mathbf{F}}_{1,2;i}=N\overline{r}{\overline{\mathbf{F}}}_{1,2}+\sum \limits_{i=1}^N\left({r}_i-\overline{r}\right)\left({\mathbf{F}}_{1,2;i}-{\overline{\mathbf{F}}}_{1,2}\right), $$

where ${\overline{r}}_{1,2}\equiv \sum \limits_{i=1}^N{r}_{1,2;i}/N$ and ${\overline{\mathbf{F}}}_{1,2}\equiv \sum \limits_{i=1}^N{\mathbf{F}}_{1,2;i}/N$. To calculate ${\overline{\mathbf{F}}}_{1,2}$, we use two assumptions: first, the elongations of the motors are comparable to their rest length, *y*_*i*_ ≈ *y*_0_; second, the unit vectors of the crosslinking protein elongations, ${\hat{\mathbf{y}}}_i$, point in a random direction with an isotropic distribution. These approximations allow us to express the average of the term in equation (8) in terms of average positions of the crosslinking proteins

11 $$ {\overline{\mathbf{F}}}_{1,2}=\frac{k}{2}\left[\begin{array}{c}\left({\overline{r}}_{2,1}-{L}_{2,1}\right)\\ {}{\overline{r}}_{2,1}\end{array}\right]. $$

Finally, the second term in Eq. (10) can be evaluated using the approximations and results given in [30],

12 $$ \sum \limits_{i=1}^N\left({r}_i-\overline{r}\right)\left({\mathbf{F}}_{1,2;i}-{\overline{\mathbf{F}}}_{1,2}\right)=\frac{1}{2{\pi}^2}\left(1+\sqrt{\frac{2\pi {k}_{\mathrm{B}}T}{k{y}_0^2}}\right)\left(\frac{1}{\sin \frac{\alpha }{2}}-\frac{1}{\cos \frac{\alpha }{2}}\right)\left[\begin{array}{c}1\\ {}1\end{array}\right], $$

where *α* is the angle between the MTs. This term is negligible in the case of motors, because it describes fluctuations around the equilibrium, which is only significant in the case for passive crosslinkers, which have no directional bias. The approach outlined here allows us to express the torque in terms of the average positions of motors or passive crosslinkers and the total number of them attached at any time, rather than positions and states of individual crosslinking proteins. In order to be able to solve the equations of motion for the MTs, we must also obtain the differential equations for those variables.

In the limit in which the number of motors in the nucleoplasm, *N*_0_, is large, the probability of finding *N* motors attached to both MTs, *p*_*N*_, is calculated using the master equation

13 $$ \frac{\mathrm{d}{p}_N}{\mathrm{d}t}={k}_{\mathrm{on}}{N}_0{p}_{N-1}+{k}_{\mathrm{off}}\left(N+1\right){p}_{N+1}-\left({k}_{\mathrm{on}}{N}_0+{k}_{\mathrm{off}}N\right){p}_N. $$

For *N* ≫ 1, *N*(*t*) can be considered a continuous variable and equation (13) can be approximated with a Langevin equation for the number of motors,

14 $$ \frac{\mathrm{d}N}{\mathrm{d}t}={k}_{\mathrm{on}}{N}_0-{k}_{\mathrm{off}}N+\sqrt{k_{\mathrm{on}}{N}_0+{k}_{\mathrm{off}}N}{\eta}_{\mathrm{N}}. $$

This equation is derived by calculating the expected value of the number of motors, $\mathrm{E}\left[N\right]={\sum}_{N=0}^{\infty }N{p}_N$ and the variance, $\operatorname{var}(N)={\sum}_{N=0}^{\infty }{N}^2{p}_N-{\left(\mathrm{E}\left[N\right]\right)}^2$ from Eq. (13). The effective number of crosslinking proteins in the nucleoplasm can be approximated by

15 $$ {k}_{\mathrm{on}}{N}_0=\frac{k_{\mathrm{on}}{c}_{\mathrm{nuc}}}{\sin \alpha }{e}^{-\frac{y_{\mathrm{min}}^2}{y_0^2}}, $$

where the constant *k*_on_*c*_nuc_ is termed the motor concentration parameter and *y*_min_ = *y*(*L*_1_, *L*_2_) is the minimal distance between the MTs.

Aside from movement of attached crosslinking proteins along MTs, motor attachment and detachment affect the average coordinates of the motors. Here it is convenient to introduce the auxiliary coordinates $\overline{u}=\left({\overline{r}}_1+{\overline{r}}_2\right)/2$ and $\overline{w}=\left({\overline{r}}_1-{\overline{r}}_2\right)/2$, because the velocities of crosslinking proteins along those coordinates are mutually independent. By considering the attachment and detachment jump processes in the continuous limit, we obtain two independent Langevin equations for the average coordinates of the motors,

16a $$ \frac{\mathrm{d}\overline{u}}{\mathrm{d}t}={v}_{\overline{\mathrm{u}}}-{k}_{\mathrm{on}}{N}_0\frac{\overline{u}-{L}_{\mathrm{u}}}{N+1}+\sqrt{\frac{k_{\mathrm{on}}{N}_0}{{\left(N+1\right)}^2}\left({\left(\overline{u}-{L}_{\mathrm{u}}\right)}^2+\frac{k_BT}{4k\ {\sin}^2\frac{\alpha }{2}}\right)}{\eta}_{\mathrm{u};\mathrm{a}}+\frac{\upsigma_{\mathrm{u}}}{\left|N-1\right|}{\eta}_{\mathrm{u};\mathrm{d}}, $$

16b $$ \frac{\mathrm{d}\overline{w}}{\mathrm{d}t}={v}_{\overline{\mathrm{w}}}-{k}_{\mathrm{on}}{N}_0\frac{\overline{w}-{L}_{\mathrm{w}}}{N+1}+\sqrt{\frac{k_{\mathrm{on}}{N}_0}{{\left(N+1\right)}^2}\left({\left(\overline{w}-{L}_{\mathrm{w}}\right)}^2+\frac{k_BT}{4k\ {\cos}^2\frac{\alpha }{2}}\right)}{\eta}_{\mathrm{w};\mathrm{a}}+\frac{\upsigma_{\mathrm{w}}}{\left|N-1\right|}{\eta}_{\mathrm{w};\mathrm{d}}, $$

where *L*_u,w_ = (*L*_1_ ± *L*_2_)/2 and ${v}_{\overline{\mathrm{u}},\overline{\mathrm{w}}}={\sum}_{i=1}^N\left({v}_{1;i}\pm {v}_{2;i}\right)/2N$ are the velocities of the average coordinates of crosslinking proteins, obtained from Eqs. (5) or (6) for motors or passive crosslinkers, respectively. The average of the force projection ${\sum}_{i=1}^N{\mathbf{f}}_{1,2;i}\bullet {\hat{\mathbf{r}}}_{1,2;i}$ is evaluated in the same way as Eqs. (11). The terms σ_u,w_ represent the standard deviations of the stationary distributions of the coordinates obtained from Eqs. (5) and (6) for motors and passive crosslinkers respectively.

Using the average coordinates given in equations (16a,b) and the number of attached motors given by Eq. (13), we calculate the torque components in Eq. (7), which determine the MT orientations, thus solving the model.

### Choice of parameter values

Our model has 12 parameters. There are 7 parameters related to motors, which we estimated based on previous in vitro measurements for kinesin-5. The movement of motors is described by their velocity at zero load, *v*_0_ =  − 0.01 μm/s, which we estimated as half the measured contour velocity (roughly −0.02 μm/s), given that the tetrameric motors walk along each MT with half of this velocity. This velocity is 3 times smaller than velocity measured from in vitro motility assays for Cut7 [23], 2 times smaller than velocity for Kip1 purified from *Saccharomyces cerevisae* from in vitro experiments, at high ionic strength conditions in [22], and 6 time smaller than velocity for purified Cin8 in budding yeast measured in [19, 21]. The motor velocity dispersion, *D*_c_ = 5 × 10^−4^μm^2^/s, is estimated based on theory (*D*_c_ = *rv*_0_*d*/2, where *d* = 36 nm is the kinesin step size and *r* ≈ 0.39 is the randomness observed in optical tweezer experiments [55]. For stall force, we used *f*_0_ =  − 1.5 pN measured for Cin8 from budding yeast [56], which is consistent with the stall force estimated for Xenopus kinesin-5 [80], but five times smaller than reported in human kinesin-5 dimeric construct [81]. The off rate 0.1 s^−1^ of motor detachment is estimated based on/from the dwell time for Cin8 [19], which is similar to the value used in [32], and two times smaller than off rate for Kip1 in [22], estimated as the motor velocity divided by its run length). Value for the motor concentration parameter is roughly estimated so that there are 10 motors attached when the MTs are in contact and the angle between them is 120°, *k*_on_*c*_nuc_ = 0.5 s^−1^. The value of 10 motors was chosen because in the experiments, at the angles in the range 90 °  − 150°, the number of Cut7 motors at the MT contact site was estimated to be of the order of 10, by using the approach from our previous work [82]. In the model, the motor is described as a Hookean spring whose stiffness is *k* = 300 pN/μm [57] and whose rest length, *y*_0_ = 53 nm, matches the length of the crossbridge, Fig. 3c in [58], which is similar to the motor rod length measured in [83, 84] and equal to the one used in [32].

For the MTs, we have 3 parameters: diffusion constant *D*_1,2_, MT length *R*_1,2_ and number of MTs in a nucleus *n*_MT_. We calculate the diffusion constant as *D* ∝ *R*^−3^ [85] using fitting results from [29], which is consistent with [30]. Our model does not include MT growth and shrinkage, which is a reasonable simplification given that MTs in this system spend most of their lifetime at a rather constant length (Additional file 1: Figure S1c) [29]. Yet, we take variability in MT length into account by assuming that MT lengths follow an exponential distribution, where the lengths of an ensemble of MTs correspond to the lengths of dynamic MTs averaged over time. The expected value of measured MT lengths is 1.5 μm [29]. We assume that the distribution of MT lengths is exponential [59], but measurements only take into account MTs longer than 0.7 μm, so the real distribution consistent with the measurements is *R*~Exp(1/0.8 μm^−1^), yielding the expected value of *R*_1,2_ = 0.8 μm. In our experiments, the average number of visible MTs on each SPB is 4, which means there are on average 10 MTs in a real cell, using the above exponential distribution. Assuming there is a roughly equal number of MTs at each SPB, this implies that there are around 25 possible combinations of MTs that can form a bundle, and the real bundling time is the fastest bundling time out of these combinations.

We varied the distance between the SPB in the range to match the variability among the cells in our experiments. The nucleus is approximated as a sphere with radius *R*_C_ = 1.5 μm, a value that is estimated from the nuclear volume [60].

### Numerical simulations

In order to obtain the average bundling times and contour velocities, we solved the system of Eqs. (7), (14), and (16a, b) numerically by simulating the sample paths. The simulations were performed using an Euler-Maruyama scheme for solving stochastic differential equations, with a reflective boundary condition representing the nuclear envelope (the envelope was assumed to be a hard spherical shell with both SPBs embedded in it). The simulations we performed slightly differently for obtaining bundling times and contour velocities.

For calculating the average bundling time, we simulated 3000 sample paths per value of *d*_SPB_ for MT angles, which lasted for *t*_max_ = 10 min or until the bundling angle between MTs of *α*_b_ = 3.05 was reached. The MT lengths were generated randomly from an exponential distribution discussed under Table 1. The initial condition for the angles was randomly generated so that the initial polar angles have a sinusoidal distribution and the azimuthal angles have a uniform distribution. If the randomly generated initial orientation would place the MT outside of the nucleus, it would be rejected and generated again. The last time of each run was recorded. In order to represent the fact that there are many MTs on each SPB, the run times were randomly organized into sets of (*n*_MT_/2)^2^, and only the fastest time would represent a single data point. This was done 10,000 times.

For simulating the contour length in time, we set the initial condition for the angles in radians (*θ*_1_, *θ*_2_, *ϕ*_1_, *ϕ*_2_)|_*t* = 0_ = (0.5, 2.6, 0.001, 0.0001), and fixed the MT length to be the same as the SPB distance, *R*_1,2_ = *d*_SPB_. We then performed 1000 runs for parameter values shown in Table 1 to obtain the sample paths. At each time point, the mean and standard deviation were calculated for all simulation runs (see Fig. 5c). The contour velocities shown in Fig. 5d were calculated by repeating the simulations for each value of the parameters of interest and performing a linear fit on the simulated data in the time interval 1 s < *t* < 5 s. The experimental velocity was obtained by performing the linear fit on all of the experimental data before the bundling time.

### Quantification and statistical analysis

*Maximum-intensity projections* were calculated with ImageJ (National Institutes of Health, Bethesda, MD, USA) using the plug-in Grouped Z Projector under Stacks selection. The color-merge images were obtained by overlay of projections in green and red channels using Merge Channels under Color selection.

*Tracking of MTs and SPBs* was done as follows: the position of MT tips was manually tracked by using Manual Tracking plug-in under Tracking selection. MT tracking was performed on MTs longer than 0.5 μm that appeared before spindle reassembly with traces longer than 1 min. MT contact was identified visually by using the tubulin signal. Note that in maximum-intensity projections, MTs may appear as in contact, while being separated along the *z*-axis. In the strains in which SPBs were fluorescently labeled, SPBs were tracked by specialized tracking software [86], whereas in other strains the position of the SPBs was estimated based on the end of MT signal.

*The average spindle reassembly time* was calculated as the total time of spindle reassembly over all cells (for non-assembled spindles this time is equal to the duration of imaging, i.e., 10 min) divided by the number of reassembly events. The error (s.e.m.) was calculated as the average reassembly time divided by the square root of the number of reassembly events.

*The contour length of MTs* was measured in 14 out of 21 cells in which MTs interacted at an oblique angle. In the remaining 7 cells, the contour length was not measured because the point of MT contact was not clearly visible in all time frames. We measured the contour length up to 10 s before and 4 s after the first antiparallel bundle was formed (time *t* = 0). We measured the contour length using Multi-point tool in Fiji. In each time frame, we measured the coordinates of three points: at one SPB, at the point of MT interaction, and at the other SPB (Additional file 1: Figure S1e). From the coordinates of these points, we calculated the angle between MTs, the contour length, and the distance between SPBs.

*Signal intensity profiles* of mCherry-tubulin and Cut7-3GFP were measured by using a Freehand Line tool in Fiji. The line was drawn starting from the cytoplasm, passing though one SPB, following the MT contour via the contact point, passing through the other SPB, and ending in the cytoplasm. A new line was drawn on each time frame in the channel for mCherry-tubulin.

Data are presented as mean ± s.e.m. The error (s.e.m.) on *proportion data* was calculated as $\sqrt{\left(p\left(1-p\right)\right)/n}$, where *n* is the sample size, and *p* is the number of events divided by *n*. Data analysis was performed using custom scripts written in MATLAB (Mathworks). Figures were assembled in Adobe Illustrator (Adobe Systems). The animation in Fig. 4c and Additional file 5: Movie S4 was generated in Wolfram Mathematica (Wolfram Research).

## Additional files

Additional file 1: **Figure S1.** Additional results on spindle reassembly. **Figure S2.** Additional time lapses and reassembly time in cells with labeled Cut7 or kinetochores. **Figure S3.** Simulations of contour length with an asymmetric initial configuration and with additional parameter variation. **Table S1.** Strains used in this study. (PDF 2070 kb)

## Abbreviations

GFP: Green fluorescent protein
MT: Microtubule
SPB: Spindle pole body
