# Supplementary material for: Pivoting of microtubules driven by minus-end-directed motors leads to spindle assembly
Source: BMC Biol. 2019 May 23;17:42. doi: 10.1186/s12915-019-0656-2 (PMC6533735; doi:10.1186/s12915-019-0656-2)
Supplement: Supplementary file 5 — Figure S1. Additional results on spindle reassembly. Figure S2. Additional time lapses and reassembly time in cells with labeled Cut7 or kinetochores. Figure S3. Simulations of contour length with an asymmetric initial configuration and with additional parameter variation. Table S1. Strains used in this study. (PDF 2070 kb) [file 12915_2019_656_MOESM1_ESM.pdf]

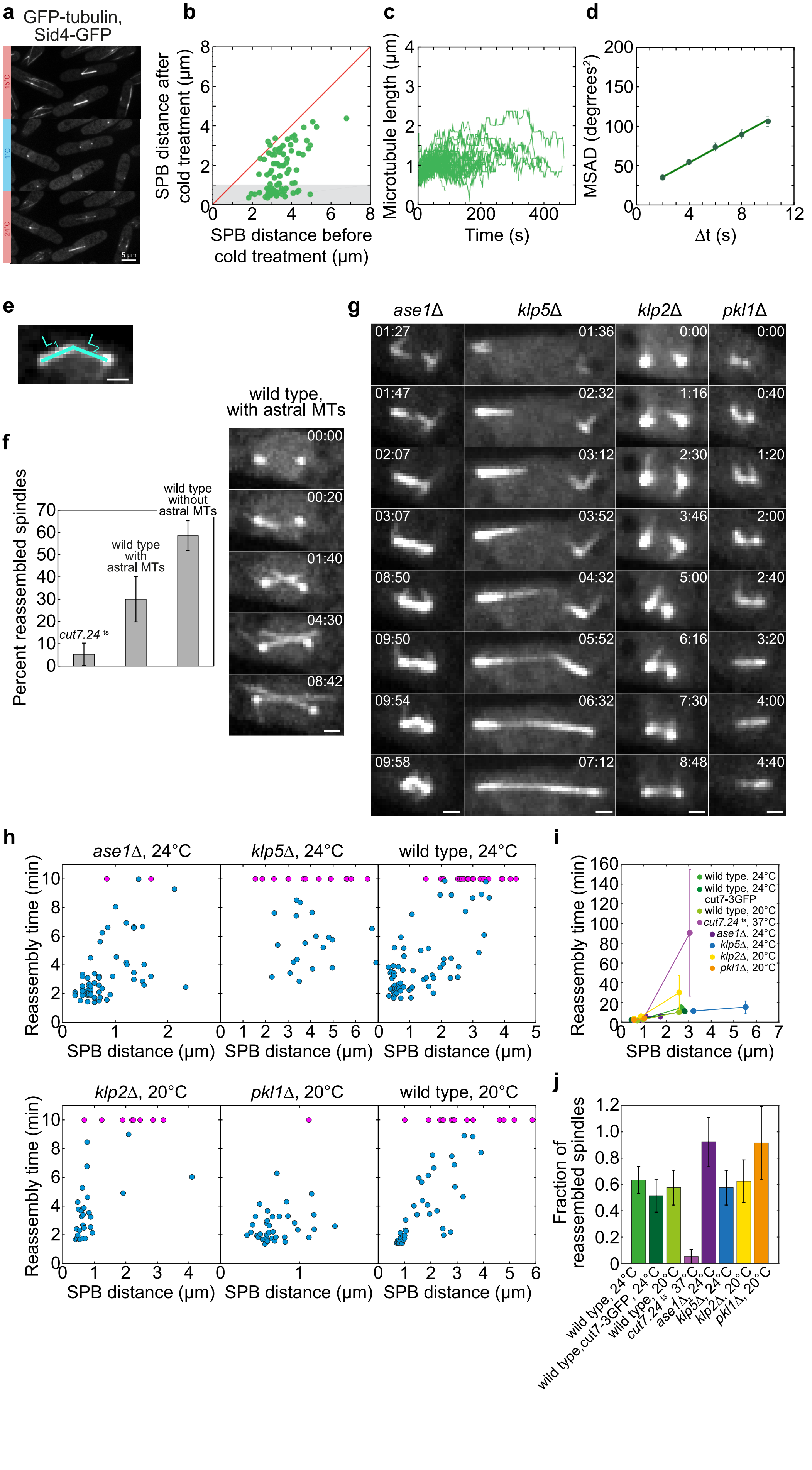

a
GFP-tubulin, Sid4-GFP
5 µm
b
SPB distance after cold treatment (µm)
SPB distance before cold treatment (µm)
c
Microtubule length (µm)
Time (s)
d
MSAD (degrees^2)
Δt (s)
e
L_1
L_2
f
Percent reassembled spindles
cut7.24 ts
wild type with astral MTs
wild type without astral MTs
wild type, with astral MTs
g
ase1Δ
klp5Δ
klp2Δ
pkl1Δ
h
ase1Δ, 24°C
klp5Δ, 24°C
wild type, 24°C
klp2Δ, 20°C
pkl1Δ, 20°C
wild type, 20°C
Reassembly time (min)
SPB distance (µm)
i
wild type, 24°C
wild type, 24°C cut7-3GFP
wild type, 20°C
cut7.24 ts, 37°C
ase1Δ, 24°C
klp5Δ, 24°C
klp2Δ, 20°C
pkl1Δ, 20°C
Reassembly time (min)
SPB distance (µm)
j
Fraction of reassembled spindles
wild type, 24°C
wild type,cut7-3GFP, 24°C
wild type, 20°C
cut7.24 ts, 37°C
ase1Δ, 24°C
klp5Δ, 24°C
klp2Δ, 20°C
pkl1Δ, 20°C

Figure S1. Additional results on spindle reassembly

a Spindle reassembly assay. A field of view showing wild-type cell expressing GFP-tubulin and Sid4-GFP (strain KI061) before (top), during (middle), and after cold treatment (bottom). Note that MTs are visible before and after, but not during cold treatment.

b Distance between the SPBs after cold treatment as a function of the distance between the SPBs before cold treatment. Note that the distance between the SPBs was smaller after than before cold treatment. In the cells in which the SPBs were more than 1 μm apart after cold treatment, it was possible to observe and quantify the process of spindle reassembly. n=84 cells; gray area denotes SPB distances smaller than 1 μm apart after cold treatment; red line is y=x.

c MT length as a function of time, n=66.

d Mean squared angular displacement (MSAD) of the MT. Data are shown as mean±s.e.m. A linear fit with weights 1/s.e.m., MSAD = $2D_{\mathrm{MT}}\Delta t$ + offset, yields D_{MT} = 4.5 degrees^2/s, where D_{MT} is the angular diffusion coefficient of the MTs. MTs of length 1-2 μm with 20 seconds long time series were used, n=598.

e Image showing how the total contour length of the MTs was measured, as a segmented line starting at one SPB, passing through the contact point between the MTs, and ending at the other SPB. The lengths of the two segments are denoted L_1 and L_2.

In panels (**b**)-(**e**), the same strain as in (**a**) were used.

f After Cut7 inactivation, i.e., at times longer than 3 minutes, the majority of these cells (17 out of 19) showed cytoplasmic astral MTs, which are characteristic for anaphase B but not earlier phases [1-3], suggesting that in this experiment the cells progressed through mitosis faster due to a higher temperature [4]. Therefore, we compared *cut7.24*^{ts} cells with wild-type cells in which cytoplasmic astral MTs were visible, and found that in the latter group 30±10% (6 out of 20) cells the spindles reassembled within 10 minutes (left panel). In *cut7.24*^{ts} cells, 5±5% (1 out of 19) spindles reassembled within 10 minutes (left panel and main text). Note that in all *cut7.24*^{ts} cells at times longer than 3 minute, and in all wild-type cells with visible cytoplasmic astral MTs, the distance between the SPBs was larger than 1 μm. Thus, we also show for comparison wild-type cells without visible cytoplasmic astral MTs in which the distance between the SPBs was larger than 1 μm (58±7%, n=31 out of 53, left panel and main text). The fraction of reassembled spindles in *cut7.24*^{ts} cells is significantly smaller than in both wild-type groups, suggesting that the unsuccessful spindle reassembly in *cut7.24*^{ts} cells was a consequence of Cut7 inactivation. Right panel shows time-lapse images of a wild-type cell expressing GFP-tubulin and Sid4-GFP (strain KI061), in which cytoplasmic astral MTs were visible after the end of cold treatment, and the spindle did not reassemble.

g Time-lapse images of mutant cells, as denoted, and **h** spindle reassembly time as a function of the distance between the SPBs at the onset of MT nucleation, for the same mutants as in (**g**), together with the corresponding wild-type cells. In (**h**), top row: *ase1*Δ (strain LW050, n=65 reassembled spindles out of 67 in total), *klp5*Δ (strain LW065, n=20 reassembled spindles out of 34), and wild-type cells (strain KI061, n=65 reassembled spindles out of 87, the same data are also shown in Fig. 1), all imaged at 24°C. Bottom row: *klp2*Δ (strain KI013, n=27 reassembled spindles out of 36), *pkl1*Δ (strain I1_2_10, n=37 reassembled spindles out of 38), and wild-type cells (strain KI061, n=39 reassembled spindles out of 53), all imaged at 20°C. Note that we did not test kinesin-6/Klp9 because deletion of this motor does not affect spindle assembly, but impairs specifically anaphase B spindle elongation, in cells that are not cold-treated [5].

i Average spindle reassembly time as a function of the distance between the SPBs for 8 data sets, each corresponding to the strain and temperature indicated in the legend. The data sets used to produce this graph are shown in panel (**h**), Fig. 3a, and Figure S2d. The SPB distances from each data set were binned into 4 bins: 0-0.75 μm, 0.75-1.5 μm, 1.5-4.5 μm, >4.5 μm. The SPB distance for each bin (x-coordinate of each circle) was calculated as the mean SPB distance of all cells in that bin. The average spindle reassembly time for each bin (y-coordinate of each circle) was calculated as the total time of spindle reassembly over all cells in that bin (for non-assembled spindles this time is equal to the duration of imaging, i.e., 10 minutes) divided by the number of reassembly events. Error bars, s.e.m.

j The fraction of spindles that reassembled within 10 minutes, for the strains and temperatures as indicated. Only the cells that reassembled spindles at times longer than 3 minutes or did not reassemble within 10 minutes were taken into account here, in order to allow for a comparison between *cut7.24*^{ts} cells and other strains.

All images are maximum-intensity projections; time is given in min:s; scale bars are 1 μm (except in panel (**a**), where the scale bar is 5 μm).

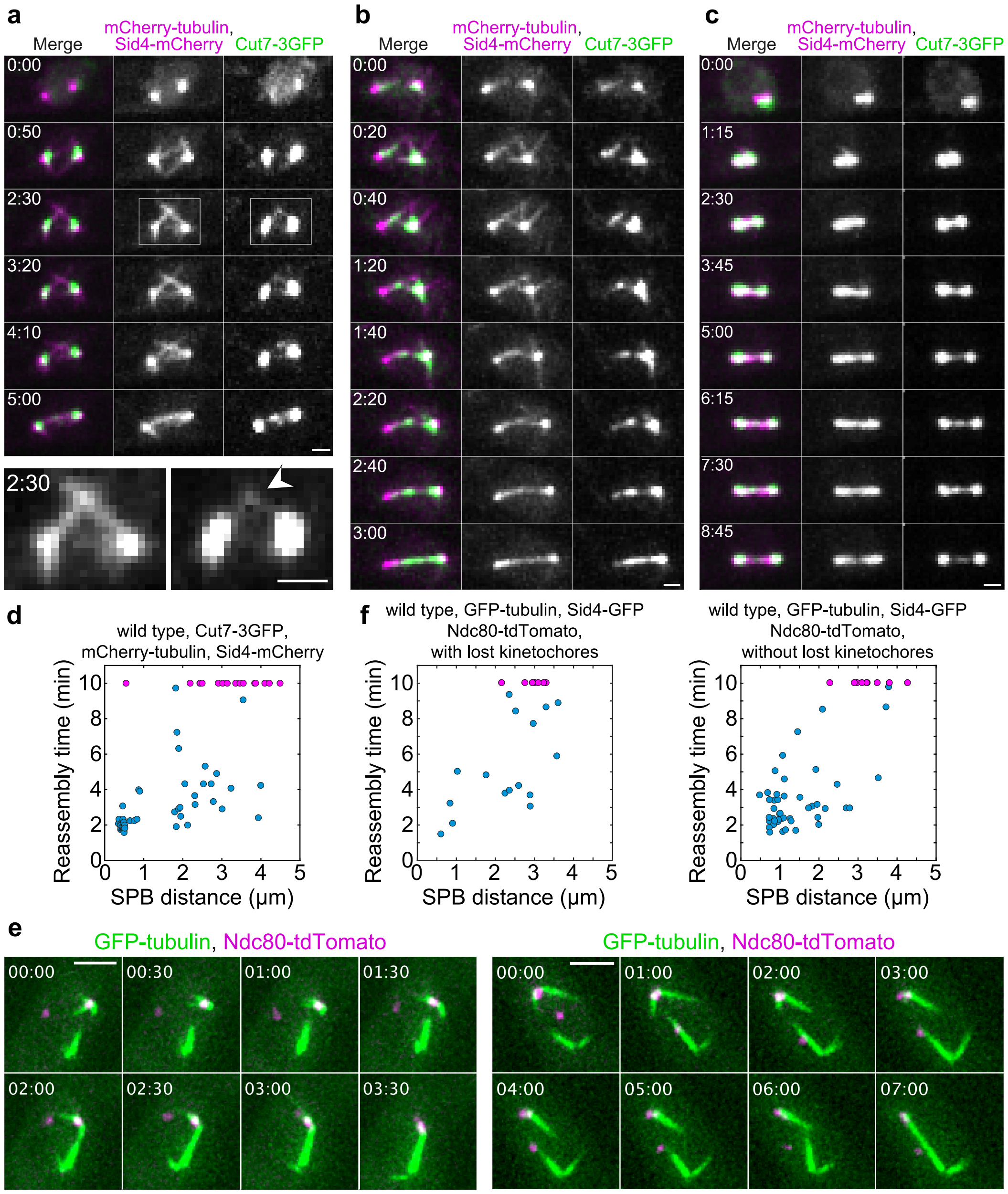
a
Merge
mCherry-tubulin, Sid4-mCherry
Cut7-3GFP
0:00
0:50
2:30
3:20
4:10
5:00
b
0:00
0:20
0:40
1:20
1:40
2:20
2:40
3:00
c
0:00
1:15
2:30
3:45
5:00
6:15
7:30
8:45
d
wild type, Cut7-3GFP, mCherry-tubulin, Sid4-mCherry
Reassembly time (min)
SPB distance (μm)
f
wild type, GFP-tubulin, Sid4-GFP Ndc80-tdTomato, with lost kinetochores
wild type, GFP-tubulin, Sid4-GFP Ndc80-tdTomato, without lost kinetochores
e
GFP-tubulin, Ndc80-tdTomato
00:00
00:30
01:00
01:30
02:00
02:30
03:00
03:30
04:00
05:00
06:00
07:00

Figure S2. Additional time lapses and reassembly time in cells with labeled Cut7 or kinetochores

a-**c** Examples of spindle reassembly in cells expressing Cut7-3GFP (green), mCherry-tubulin (magenta), and Sid4-mCherry (magenta; strain LW042). Merged time-lapse images (left column) and separate channels (central and right column, both in gray scale) are shown. In (**a**) at the bottom, enlargements of the spindle in the boxed region at 2:30 min show the accumulation of Cut7 (arrowhead) at the site of initial contact of MTs extending from the two SPBs. In (**b**), Cut7 accumulates at the site of initial contact of MTs at 1:20 min. Note that the cell in (**a**) is an outlier with respect to the time elapsed from Cut7 appearance at the MT contact point until MT alignment into an antiparallel configuration (2.5 minutes), whereas the typical time was less than 1 minute. In (**c**), the initial distance between the SPBs is smaller than 1 μm, thus it was not possible to observe the initial MT contact. Images are maximum-intensity projections, time is given in min:s; scale bars, 1 μm.

d Spindle reassembly time as a function of the distance between the SPBs at the onset of MT nucleation, for the same strain as in (**a**)-(**c**), imaged at 24°C (n=45 reassembled spindles out of 61).

e Two examples of spindle reassembly in cells expressing GFP-tubulin (green) and Ndc80-tdTomato (a kinetochore marker, magenta; strain AH01). Note that spindle reassembly including MT alignment occurs without kinetochores being present close to the MT contact point. In these experiments, cold treatment was performed as described in [6], and images were acquired by using a DeltaVision RT system. Time is given in min:s; scale bars, 2 μm.

f Spindle reassembly time as a function of the distance between the SPBs at the onset of MT nucleation, for the cells expressing GFP-tubulin, Sid4-GFP, and Ndc80-tdTomato (strain KI061), in which kinetochores were imaged after the cold treatment. Left, only the cells that have a lost kinetochore, i.e., a free kinetochore in the nucleoplasm (n=16 reassembled spindles out of 23). Right, the cells without lost kinetochores (n=46 reassembled spindles out of 55).

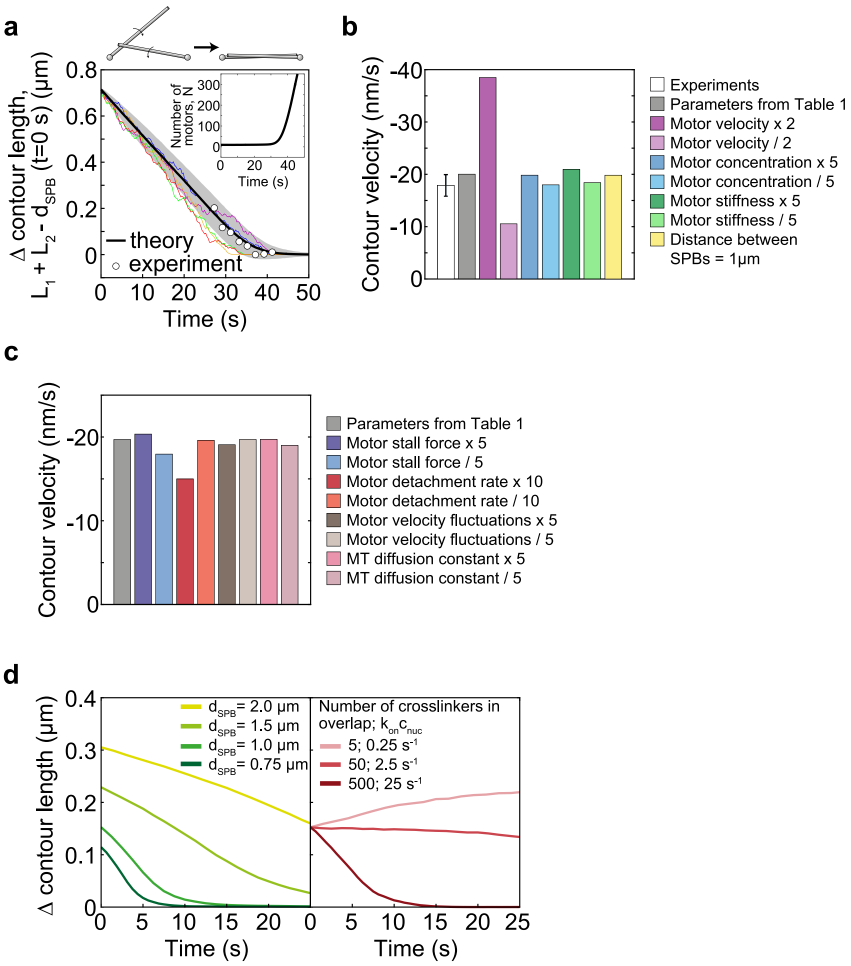

Figure S3. Simulations of contour length with an asymmetric initial configuration and with additional parameter variation

a Contour length difference (main graph) and the number of motors (inset) as a function of time. In the simulations, MTs start from the asymmetric configuration where the initial angles are $\theta_1 = 60\,°$ and $\theta_2 = 150\,°$. The minimal distance between the MTs is negligible, $y_{\text{min}} \approx 0$. We define the contour length as the sum of the lengths L_1 and L_2, which extend from the SPB to the point on the MT that is closest to the other MT, and the contour length difference is calculated as $L_1 + L_2 - d_{\text{SPB}}$. The black line and the shaded area represent the average value and standard deviation, respectively, for all simulation results at a given time point. The colored lines are sample paths for simulations. The white dots represent the mean experimental values shown in Fig. 2d, with their origin on the time axis shifted by the average bundling time of the simulation runs. Simulations are performed with the parameters form Table 1, $d_{\text{SPB}} = 2$ μm and $R_{1,2} = 2$ μm.

b Contour velocity in experiments (white bar; error bar is s.e.m.) and in simulations for different parameter values (colored bars). Deviations from parameter values in Table 1 and $d_{\text{SPB}} = 2$ μm are denoted in the legend. MT lengths are $R_{1,2} = 2$ μm, except for the last bar, where $R_{1,2} = 1$ μm. Initial configurations are as in (**a**).

c Contour velocity in simulations starting form a symmetrical configuration for different parameter values. Deviations from parameter values used in Table 1 are denoted in the legend; $d_{\text{SPB}} = 2$ μm and $R_{1,2} = 2$ μm.

d Contour length difference as a function of time for different SPB distances (left) and for different concentration of passive crosslinkers (right), obtained from the model. The green lines represent the average value for varied SPB distances shown in the legend and linker concentration parameter 25 s^{-1} (500 passive crosslinkers in the overlap). The red lines represent the average value for different number of crosslinkers in overlap (different crosslinker concentration) shown in the legend and $d_{\text{SPB}} = 1$ μm. Initial configuration is symmetrical and parameters taken from Table 1 except for those that differ between motors and passive crosslinkers: diffusion constant $D_{\text{c}} = 0.05\ \mu\text{m}^2/\text{s}$ [7], and the rest length $y_0 = 40$ nm [8].

Table S1. Strains used in this study

Name	Genotype	Source
KI061	*h⁻ cdc25-22 ndc80-nmtP41-tdTomato-kanMX6 kan^r-nmtP3-GFP-atb2$^+$ sid4-GFP-kan^r*	lab stock [6]
AH01	*h$^+$ cdc25-22 ndc80-nmtP41-tdTomato-kanMX6 kan^r-nmtP3-GFP-atb2$^+$*	lab stock [6]
CF.391	*h$^+$ cut7.24 cdc13-GFP:nat^r mCherry-atb2:hyg^r ade6-m210? leu1-32 ura4-D18*	Phong Tran
#3208	*h$^+$ ase1::hyg^r ura4-D18 leu1-32*	Jonathan Millar
LW050	*h$^+$ cdc25-22 ndc80-nmtP41-tdTomato-kanMX6 kan^r-nmtP3-GFP-atb2$^+$ ase1::hyg^r*	This study
LW065	*h$^+$ cdc25-22 ndc80-nmtP41-tdTomato-kanMX6 kan^r-nmtP3-GFP-atb2$^+$ klp5Δ*	This study
I1_2_10	*nmtP3-GFP-atb2$^+$ pkl1Δ*	This study
KI013	*ndc80-nmtP41-tdTomato-kanMX6 kan^r-nmtP3-GFP-atb2$^+$ sid4-GFP-kan^r klp2Δ*	lab stock
SI661'	*h$^+$ sid4$^+$-mCherry*	lab stock
PT.2973	*h$^-$ cut7-3xGFP mCherry-atb2:hyg^r ade6-m210? leu1-32 ura4-D18*	Phong Tran
LW042	*h$^+$ cut7-3xGFP mCherry-atb2:hyg^r ade6-m210? leu1-32 ura4-D18 sid4$^+$-mCherry*	This study

References

1. Sagolla MJ, Uzawa S, Cande WZ. Individual microtubule dynamics contribute to the function of mitotic and cytoplasmic arrays in fission yeast. J Cell Sci 2003;116(Pt 24):4891-4903.
2. Zimmerman S, Daga RR, Chang F. Intra-nuclear microtubules and a mitotic spindle orientation checkpoint. Nat Cell Biol 2004;6(12):1245-1246.
3. Vogel SK, Raabe I, Dereli A, Maghelli N, Tolic-Norrelykke I. Interphase microtubules determine the initial alignment of the mitotic spindle. Curr Biol 2007;17(5):438-444.
4. Nabeshima K, Nakagawa T, Straight AF, Murray A, Chikashige Y, Yamashita YM, Hiraoka Y, Yanagida M. Dynamics of centromeres during metaphase-anaphase transition in fission yeast: Dis1 is implicated in force balance in metaphase bipolar spindle. Mol Biol Cell 1998;9(11):3211-3225.
5. Fu CH, Ward JJ, Loiodice I, Velve-Casquillas G, Nedelec FJ, Tran PT. Phospho-Regulated Interaction between Kinesin-6 Klp9p and Microtubule Bundler Ase1p Promotes Spindle Elongation. Dev Cell 2009;17(2):257-267.
6. Kalinina I, Nandi A, Delivani P, Chacon MR, Klemm AH, Ramunno-Johnson D, Krull A, Lindner B, Pavin N, Tolic-Norrelykke IM. Pivoting of microtubules around the spindle pole accelerates kinetochore capture. Nat Cell Biol 2013;15(1):82-87.
7. Kapitein LC, Janson ME, van den Wildenberg SM, Hoogenraad CC, Schmidt CF, Peterman EJ. Microtubule-driven multimerization recruits ase1p onto overlapping microtubules. Curr Biol 2008;18(21):1713-1717.
8. Kellogg EH, Howes S, Ti SC, Ramirez-Aportela E, Kapoor TM, Chacon P, Nogales E. Near-atomic cryo-EM structure of PRC1 bound to the microtubule. Proc Natl Acad Sci U S A 2016;113(34):9430-9439.
